# The crystal structure of the killer fibre erionite from Tuzköy (Cappadocia, Turkey)

**DOI:** 10.1107/S2052252523003500

**Published:** 2023-05-18

**Authors:** Carlotta Giacobbe, Anna Moliterni, Dario Di Giuseppe, Daniele Malferrari, Jonathan P. Wright, Michele Mattioli, Simona Raneri, Cinzia Giannini, Laura Fornasini, Enrico Mugnaioli, Paolo Ballirano, Alessandro F. Gualtieri

**Affiliations:** a European Synchrotron Radiation Facility (ESRF), 71 avenue des Martyrs, Grenoble 38000, France; b Institute of Crystallography-CNR, Via Amendola 122/o, Bari 70126, Italy; c Dipartimento di Scienze Chimiche e Geologiche, Università degli Studi di Modena e Reggio Emilia, Via G. Campi 103, Modena 41125, Italy; d Dipartimento di Scienze Pure ed Applicate, Università degli Studi di Urbino Carlo Bo, Campus Scientifico Enrico Mattei, Urbino 61029, Italy; e ICCOM-CNR, Institute of Chemistry of Organometallic Compounds, Italian National Research Council, Via G. Moruzzi 1, Pisa 56124, Italy; f Dipartimento di Scienze della Terra, Università di Pisa, Via S. Maria 53, Pisa 56126, Italy; g Dipartimento di Scienze della Terra, Sapienza - Università di Roma, Piazzale Aldo Moro 5, Roma 00185, Italy; SPring-8, Japan

**Keywords:** erionite, nano-diffraction, asbestos, *ab initio* structure solution, mesothelioma

## Abstract

Erionite is a non-asbestos fibrous zeolite classified by the International Agency for Research on Cancer (IARC) as a Group 1 carcinogen and is considered today similar to or even more carcinogenic than the six regulated asbestos minerals. Exposure to fibrous erionite has been unequivocally linked to cases of malignant mesothelioma (MM) and this killer fibre is assumed to be directly responsible for more than 50% of all deaths in the population of the villages of Karain and Tuzköy in central Anatolia (Turkey). Erionite usually occurs in bundles of thin fibres and very rarely as single acicular or needle-like fibres. For this reason, a crystal structure of this fibre has not been attempted to date although an accurate characterization of its crystal structure is of paramount importance for our understanding of the toxicity and carcinogenicity. In this work, we report on a combined approach of microscopic (SEM, TEM, electron diffraction), spectroscopic (micro-Raman) and chemical techniques with synchrotron nano-single-crystal diffraction that allowed us to obtain the first reliable *ab initio* crystal structure of this killer zeolite. The refined structure showed regular *T*—O distances (in the range 1.61–1.65 Å) and extra-framework content in line with the chemical formula (K_2.63_Ca_1_._57_Mg_0.76_Na_0.13_Ba_0.01_)[Si_28.62_Al_7.35_]O_72_·28.3H_2_O. The synchrotron nano-diffraction data combined with three-dimensional electron diffraction (3DED) allowed us to unequivocally rule out the presence of offretite. These results are of paramount importance for understanding the mechanisms by which erionite induces toxic damage and for confirming the physical similarities with asbestos fibres.

## Abstract 2

Synchrotron nano-diffraction coupled with electron diffraction and spectroscopic techniques revealed the crystal structure of zeolite erionite from Cappadocia. These results fill in the gap for precise modelling of the carcinogenicity of this killer fibre.

## 1. Introduction

**Figure 1:**
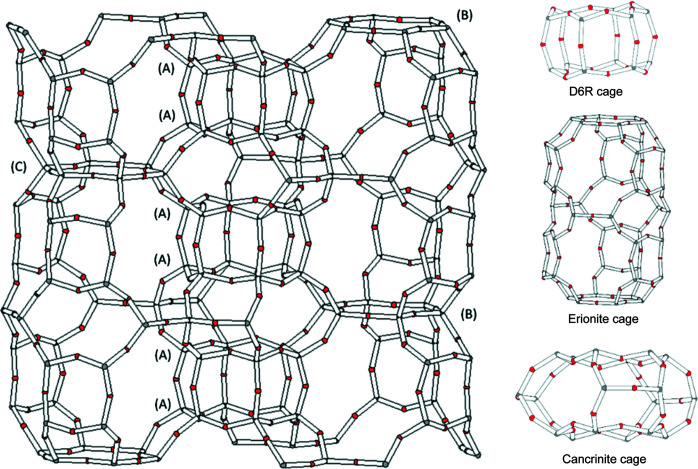
Erionite structure consists of (A) 6-ring (D6R) cages, (B) columns of erionite cavities and (C) cancrinite (ɛ) cages.

Erionite is a natural zeolite that belongs to the ABC-6 family (Gottardi & Galli, 1985 ▸). Its periodic building unit (PerBU) consists of a hexagonal array of planar six-membered rings of (Si, Al)O_4_ tetrahedra (T6-rings) related by pure translations along *a* and *b* (Van Koningsveld, 2007 ▸). Erionite has the average formula K_2_(Ca_0.5_,Na)_7_[Al_9_Si_27_O_72_]·28H_2_O (Passaglia & Sheppard, 2001 ▸) and hexagonal symmetry with the space group *P*6_3_/*mmc* and unit-cell parameters *a* ≃ 13.15 Å, *c* ≃ 15.05 Å. In the stacking sequence of the erionite framework, neighbouring T6-rings are connected through tilted 4-rings along [001] following the AABAAC… arrangement. This three-dimensional framework is defined by columns of cancrinite (ɛ) cages ([4^6^6^5^] polyhedra) connected with double 6-ring (D6R) cages (hexagonal prism, [4^6^6^2^] polyhedron, formed by two ‘A’ 6-rings) and columns of erionite cavities ([4^12^6^5^8^6^] polyhedral) between the ‘B’ or ‘C’ 6-rings (see Fig. 1 ▸; Staples & Gard, 1959 ▸; Kawahara & Curien, 1969 ▸; McCusker *et al.*, 2001 ▸; Deer *et al.*, 2004 ▸; Ballirano & Cametti, 2012 ▸). Adjacent ɛ cages are alternately rotated by 60°.

Erionite may occur as a diagenetic product or as a result of hydro­thermal alteration in volcanic rock (Passaglia *et al.*, 1998 ▸). A large chemical variability is typical of this mineral and, according to the most abundant extra-framework cation, three different species of erionite have been identified: erionite-Na, erionite-K and erionite-Ca (Coombs *et al.*, 1997 ▸; Passaglia *et al.*, 1998 ▸; Gualtieri *et al.*, 1998 ▸; Dogan & Dogan, 2008 ▸). Previous structural refinements of erionite samples indicate that K^+^ cations are located at the centre of the ɛ cages (Gualtieri *et al.*, 1998 ▸). Other cations (Na^+^, Ca^+2^ and Mg^2+^) and water molecules occupy the erionite cages and are distributed on sites located on the symmetry axis (Gualtieri *et al.*, 1998 ▸). Alberti *et al.* (1997 ▸) found that there are three partially occupied positions (Ca1, Ca2 and Ca3) in the erionite cages and each is coordinated with water molecules. One additional cationic site was observed at a special position (1/2, 0, 0) by Ballirano *et al.* (2009 ▸) in erionite-K and labelled K2. This site corresponds to the K site found by Schlenker *et al.* (1977 ▸) in dehydrated erionite-Ca and to the Ca4 site found by Gualtieri *et al.* (1998 ▸) in some natural erionite-Ca samples and was attributed to the presence of extra-framework K^+^, Na^+^ and Ca^2+^ (Ballirano *et al.*, 2017 ▸).

The growth of the erionite crystals along the *c* axis gives it an elongated morphology that often develops into an asbestos-like habit (Belluso *et al.*, 2017 ▸). As provided by long-term epidemiological studies and several *in vivo* tests, fibrous erionite is responsible for epidemics of malignant mesothelioma (MM) in Cappadocia (Turkey), where three villages (Karain, Tuzköy and Sarihidir) were built with erionite-bearing tuff rocks (Carbone *et al.*, 2007 ▸; IARC, 2012 ▸). MM is a highly aggressive cancer that arises from mesothelial cells of the pleura, peritoneum and pericardium, with a median survival of about a year from diagnosis (Carbone & Yang, 2012 ▸). The epidemic of MM in Cappadocia was first described by Baris *et al.* (1978 ▸), but epidemiological studies of how and why this disease occurs in different groups of people in Cappadocia are still ongoing (Bariş *et al.*, 1996 ▸; Metintaş *et al.*, 2017 ▸). The data acquired to date show that fibrous erionite is identified as the cause of MM in over 50% of the population in the three aforementioned villages (Dogan, 2012 ▸). In Tuzköy, the annual incidence of MM was estimated at 22 cases per 10000, it was identical for men and women and the mean age was roughly 50, with the range 26–75 years (Artvinli & Bariş, 1979 ▸; Baris *et al.*, 1978 ▸; Simonato *et al.*, 1989 ▸; Carbone *et al.*, 2007 ▸; Emri, 2017 ▸). Traces of erionite have been detected in the air and lungs of people from these villages and it has been suggested that inhalation of even small amounts of erionite is sufficient to cause MM (Baris *et al.*, 1981 ▸; Sebastien *et al.*, 1981 ▸; Carbone *et al.*, 2007 ▸). More recently, erionite exposure issues have also emerged in the USA (Van Gosen *et al.*, 2013 ▸). For these reasons, the International Agency for Research on Cancer (IARC) included fibrous erionite as a Group 1 ‘substance carcinogenic to humans’ (IARC, 2012 ▸), and this mineral is considered today similar to or even more carcinogenic than the six regulated asbestos minerals (Wylie, 2017 ▸).

Although the toxic and carcinogenic potential of fibrous erionite is not in question, the mechanisms by which it induces cyto- and genotoxic damage are not yet fully understood (Gualtieri *et al.*, 2016 ▸). Carcinogenesis is a complex phenomenon and multiple factors can contribute to erionite toxicity and MM development: (1) the morphology of erionite fibres. As shown in the literature data, length (*L*) and width (*W*) of fibres are key factors in the toxicity, inflammation and pathogenicity of asbestos and erionite fibres (Donaldson *et al.*, 2010 ▸; Gualtieri, 2018 ▸; Carbone *et al.*, 2019 ▸). Fibres with high *L*/*W* ratios can reach the low respiratory tract of the lungs and their retention in the parietal pleura leads to the initiation of inflammation and pleural pathology such as MM (Donaldson *et al.*, 2010 ▸; Carbone *et al.*, 2019 ▸). Fibrous particles with *L* > 8 µm and *W* < 5 µm cannot be eliminated by macrophages (Churg, 1993 ▸), leading to an inflammation process known as ‘frustrated phagocytosis’ (Gualtieri, 2018 ▸). (2) Genetics predisposition. Recent studies showed that specific genetic mutations of mesothelial cells (*i.e.* germline BAP1 mutations) increase the susceptibility of developing MM at very low levels of erionite exposure (Carbone *et al.*, 2013 ▸, 2019 ▸). (3) The iron-bearing particles present at the erionite surface. These impurities may be responsible for carcinogenic activity namely via free radical production (Gualtieri *et al.*, 2016 ▸). According to the model described by Gualtieri *et al.* (2016 ▸), surface particles may dissolve during phagocytosis when the erionite fibres are engulfed in the phago-lysosome sacks at pH = 4–4.5. Dissolution may leave a residue of iron atoms at specific sites anchored to the surface windows of the six-membered rings. These surface sites can be responsible for producing H_2_O_2_ that accounts for adverse effects at the cellular and subcellular levels. (4) The high biopersistence of erionite fibres, *i.e.* their ability to remain in the human body despite physicochemical processes such as dissolution, leaching, breaking, splitting or mechanical clearance. Acid zeolites like erionite with an Si/Al ratio > 2.5 are insoluble in an acidic environment (like the extracellular environment of the lung) because the expected dealumination process does not lead to a collapse of the framework (Gualtieri *et al.*, 2018 ▸). (5) The cation-exchange capacity of erionite. In contact with the extracellular and intracellular solutions, erionite fibres may induce ion exchange with the release of extra-framework metals in the lung environment (Gualtieri *et al.*, 2019 ▸) and alteration of the cell homeostasis (Di Giuseppe *et al.*, 2022 ▸).

In the literature, many authors reported the mineralogical and chemical characterization of a wide range of erionite samples [see for example, Ballirano *et al.* (2017 ▸) and references therein] to try to correlate the toxicity/pathogenicity mechanisms of fibrous erionite to its mineralogical and microstructural properties. However, until now the crystal structure of this carcinogenic fibrous erionite from the three villages of the Cappadocia region has not been determined. We report the crystal structure of a single erionite fibre from Tuzköy village, determined by synchrotron nano-diffraction. A detailed crystal structure investigation of this zeolite is a fundamental step needed to draw a general model of the toxicity/carcinogenicity of Turkish’s fibrous erionite and elucidate the trigger mechanisms of MM.

## 2. Experimental

### 2.1. Geological overview

The fibrous erionite selected for the study is from Tuzköy, a village located at the junction of the Derinoz and Kizilirmak Rivers in the Nevşehir province (Cappadocia, Turkey). A detailed geological description of this area is reported by Temel & Gündoğdu (1996 ▸). Tuzköy village is situated near an erionite-bearing outcrop of the Zelve ignimbrite unit (upper Miocene). This geological unit is characterized by a basal pumice fall layer and was formed by several pyroclastic units that cover an area of about 4200 km^2^ in the north of Nevsehir (Temel & Gündoğdu, 1996 ▸). Erionite has crystallized with other zeolites (mainly clinoptilolite and chabazite) through hydration reactions (diagenetic processes) from the amorphous aluminosilicate glass of the pyroclastic material deposited in alkaline and saline environments (Temel & Gündoğdu, 1996 ▸; Dogan, 2003 ▸).

### 2.2. Thermogravimetric and differential thermal analysis

The evolution of the release of volatiles from the samples was analysed by thermo-gravimetric and differential thermal analysis (TG-DTA) using a simultaneous differential thermal analysis (SDTA) SEIKO SSC/5200 SII instrument. Data were collected in air with a flow rate of 2 µl min^−1^ in the range 27–1112 °C, and with a heating rate of 10 °C min^−1^.

### 2.3. Micro-Raman analysis

The sample of erionite from Tuzköy (Turkey) was analysed by µ-Raman spectroscopy. The fibrous powdered sample was prepared for the analysis in a sealed configuration, working in a fume hood: a small amount of powder (≤1 mg) was stuck to a double-sided tape adhering to a microscope glass. The powder was covered by a coverslip and sealed to the glass substrate. Fibrous erionite from Jersey (Nevada, USA) was analysed in the same sealed configuration as the reference. An offretite crystal from Saviore dell’Adamello, Brescia (Italy) was used to discriminate any presence of offretite in the sample.

The µ-Raman measurements were performed with two spectrometers. The fibrous content was examined with a HORIBA Jobin Yvon LabRam HR Evolution confocal micro-spectrometer (800 mm focal length) using an He–Ne 632.8 nm laser line as the excitation source, with an integrated Olympus BX41 microscope with 5×, 10×, 50× ULWD and 100× objectives, a 600 grooves mm^−1^ grating, an *XYZ* motorized stage and liquid-nitro­gen-cooled silicon CCD. The pinhole was fixed at 100 µm to reduce the upper glass contribution. The spectral resolution is about 2 cm^−1^. Minor mineral phases and iron-containing compounds in the sample were analysed with a HORIBA Jobin Yvon LabRam confocal micro-spectrometer (300 mm focal length) using an He–Ne 632.8 nm laser line as the excitation source, with an integrated Olympus BX40 microscope with 4×, 10×, 50× ULWD and 100× objectives, an 1800 grooves mm^−1^ grating, an *XY* motorized stage and a Peltier cooled silicon CCD. The spectral resolution is about 2 cm^−1^. The systems were calibrated using the 520.6 cm^−1^ Raman peak of silicon. The spectra were recorded in the 100–1200 cm^−1^ spectral region with typical exposures of 30 s repeated at least ten times. Data analysis was performed by the *LabSpec 5* built-in software. Fits with band deconvolution were carried out using Gauss–Lorentzian functions.

### 2.4. X-ray powder diffraction

The fibrous erionite sample studied in this work was extracted from a friable yellowish tuff in which the crystal fibres were not visible to the naked eye. To obtain the full mineralogical composition of the whole tuff, a representative sample was ground with ethanol to prevent the fibres from dispersing, and the resulting powder was homogenized in an agate mortar. The mineralogical composition of the sample was determined by X-ray powder diffraction (XRPD). The pattern was collected using a conventional Bragg–Brentano Philips diffractometer (model PW-1729), with θ–2θ geometry, Cu *K*α radiation, 40 kV, 30 mA and a KSA Energy dispersive detector. The powder was loaded on an aluminium sample holder. Data were collected in continuous mode with a 2 mm fixed divergence and anti-scatter slits mounted in the incident beam. An integrated step-scan of the detector of 0.02 °2θ was used with a time of 25 s, from 3 to 55 °2θ. Phase identification was performed using the search–match procedure developed in *Match!* (Crystal Impact, 2014 ▸). Instrument parameters and crystal structure data for each phase present in the samples were input into the *TOPAS5* suite (Coelho, 2018 ▸). The background coefficient (polynomial function), lattice parameters, zero-shift error, scale factor, peak shape parameters and absorption coefficient were refined.

### 2.5. Scanning electron microscopy

Qualitative observations of mineral fibres inside the sample were performed by scanning electron microscopy (SEM). SEM analyses were performed using an FEI Nova NanoSEM 450 FEG-SEM equipped with an X-EDS Bruker QUANTAX-200 system, with 20 kV accelerating voltage, 5 mm working distance and 3.5 µA beam current. A small amount of unground sample was mixed with 1 ml of water. A drop of the suspensions was laid on a carbon tape mounted on an Al stub, left to air dry and gold-coated (10 nm thick). Images were collected using the signal of both back-scattered and secondary electrons. The surface of the samples was investigated, working at different magnification levels. The length (*L*) and width (*W*) of fibres were determined on about 110 individual particles, using 30 SEM images. *L* and *W* were calculated using the *ImageJ* image analysis software (version 1.52*a*; Rasband, 1997–2018 ▸). Energy-dispersive X-ray spectroscopy (X-EDS) data were collected for the qualitative determination of the chemical composition of erionite fibres.

### 2.6. Transmission electron microscopy

Three dimensional-electron diffraction (3DED) data (Kolb *et al.*, 2007 ▸; Mugnaioli & Gemmi, 2018 ▸; Gemmi *et al.*, 2019 ▸) were collected with a Zeiss Libra TEM operating at 120 kV and equipped with an LaB_6_ source. 3DED acquisitions were performed in STEM mode after defocusing the beam to give a pseudo-parallel illumination on the sample. A beam size of about 150 nm in diameter was obtained by inserting a 5 µm C2 condenser aperture. An extremely mild illumination was adopted to avoid any alteration or amorphization of the sample.

3DED data were collected in discrete steps of 1° on ten crystals that were identified by energy-dispersive X-ray spectroscopy (EDS). To reduce dynamical effects, the data for the structure solution of erionite were taken using beam precession with an inclination angle of 1° (Vincent & Midgley, 1994 ▸; Mugnaioli *et al.*, 2009 ▸), obtained by a Nanomegas Digistar P1000 device. The best-3DED dataset on erionite included a total tilt range of 95°. The camera length was 180 mm, with a theoretical resolution limit of 0.75 Å. ED data were recorded by an ASI Timepix detector, which records the arrival of single electrons and delivers a pattern that is virtually background-free. Data were analysed by *ADT3D* (Kolb *et al.*, 2011 ▸) for cell and space group determination and by *PETS2* (Palatinus *et al.*, 2019 ▸) for intensity integration. *Ab initio* structure determination was obtained by direct methods implemented in the software *SIR2014* (Burla *et al.*, 2015 ▸). Data were treated with the kinematical approximation (*I*_*hkl*_ ∝ *F* ^2^_*hkl*_).

### 2.7. Quantitative chemical analysis

Quantitative chemical composition of the fibrous erionite was obtained at the Department of Earth Sciences, University of Milan, using a Jeol 8200 SuperProbe Electron Probe Microanalyzer equipped with a wavelength-dispersive X-ray (WDS) spectrometer system, W hairpin-type filament. The detectable wavelength is 0.087–9.3 nm. The atomic number resolution on BSE is (*Z*) ≤ 0.1 (Cu*Z*). The following analytical conditions were used: 15 kV excitation voltage, 5 nA specimen current, 30 s peak-count time and 10 s background-count time. The instrument was also equipped with an EDX system characterized by a detectable element range: Na to U, energy resolution: 144 eV and lithium (Li)-doped silicon single-crystal semiconductor detector. The following elements were measured at each analytical spot: Si, Al, Mg, Ca, Na, K, Fe and Ba. Calibration used a set of standards: omphacite for Na; orthoclase for K; forsterite for Mg; fayalite for Fe; and grossular garnet for Al, Si, and Ca. The raw data were corrected for matrix effects using the phi-rho-Z method from the Jeol series of programs.

### 2.8. Nano-single-crystal diffraction

Erionite fibres were manually separated from the tuff matrix under a stereoscopic optical microscope. Several crystals were glued onto MiTeGen microloops (Fig. S1 of the supporting information) and mounted on magnetic supports compatible with the nanoscope station at ID11 (Wright *et al.*, 2020 ▸; Giacobbe *et al.*, 2021 ▸). Nano-single-crystal X-ray diffraction (n-SCXRD) datasets of the erionite fibre (∼350 × 540 nm × 20 µm, see Fig. S2) were collected at beamline ID11 [The European Synchrotron Radiation Facility (ESRF), Grenoble, France] using a monochromatic beam produced by a bent Si(111) Laue–Laue double-crystal monochromator (38 keV, wavelength λ = 0.3257 Å, relative bandwidth Δλ/λ ≃ 10^−3^). Beam damage tests due to possible beam heating were performed before the data collection (Lawrence-Bright *et al.*, 2021 ▸). The diffraction images were collected with a sample-to-detector distance of 118.81 mm using a Dectris Photon Counting Eiger2 4M CdTe detector with an array of 2162 × 2068 pixels of 75 × 75 µm. Precise calibration of the detector distance and tilts was obtained using a crystalline CeO_2_ standard and the spatial alignment of the detector modules was determined using the novel procedure described elsewhere (Wright *et al.*, 2022 ▸). Diffraction frames were collected with a continuous scan over 360° (slicing 0.1°) on the most suitable erionite crystal. Three datasets were measured in different positions of the fibre. NeXus/HDF5 data were then converted into the ‘Esperanto’ format using the script *Eiger2crysalis*, a portable image converter based on the FabIO library to export Eiger frames (including those from LImA) to a set of Esperanto frames which can be imported into *CrysalisPro* (Rigaku, 2015 ▸). The converted images were successively indexed and integrated using *CrysAlisPro*.

Absorption effects were corrected using *SCALE3 ABSPACK* of *CrysAlis* (Rigaku, 2015 ▸) via a multi-scan semi-empirical approach. *R*_int_ values of 5.4% were obtained (with a data resolution of 0.75 Å). The crystal structure was solved by direct methods using *SIR2019* (Burla *et al.*, 2015 ▸) and refined using *SHELXL*-2014 (Sheldrick, 2015 ▸). The material for publication was prepared by *WinGX* (Farrugia, 2012 ▸) and *publCIF* (Westrip, 2010 ▸). The CheckCIF procedure available at *https://checkcif.iucr.org/* was used to validate the model.

## 3. Results

### 3.1. TG-DTA

Thermal analysis of the bulk sample is reported in Fig. S3. The sample exhibits three main endothermic events which occur with maximum reaction rates at about 72.7, 149, 657 and 830 °C. The weight losses corresponding to the first two thermal events are 1.46 and 3.88%, respectively. These thermal events are related to the release of water that, as typical in many zeolites or zeolite-based materials, begins from early heating stages (Scapino *et al.*, 2017 ▸; Arletti *et al.*, 2018 ▸). The third main thermal event around 657 °C (8.43% weight loss) is related to de­hydroxy­lation reactions (Ward, 1972 ▸). The fourth event at 830 °C corresponds to the release of CO_2_ following the decomposition of carbonates (Mertens *et al.*, 2007 ▸).

### 3.2. Raman spectroscopy

The Raman spectra of the erionite from Tuzköy and the erionite reference are shown in Fig. S4. Although natural erionite and offretite can intergrow, the presence of offretite was ruled out in the sample from Tuzköy, as µ-Raman analysis can reliably assess the presence/absence of the two phases. Raman spectra acquired on the fibres correspond to the zeolite erionite, while the offretite contribution was not detected (the characteristic peak of offretite at 431 cm^−1^ is absent). The Raman spectrum of an offretite crystal with its distinctive habit from a typical locality where this zeolite was found (Saviore dell’Adamello, Brescia, Italy) is also reported in Fig. S4 (Passaglia *et al.*, 1998 ▸; Guastoni *et al.*, 2002 ▸).

The main Raman features of erionite are observed in the spectral region between 400 and 600 cm^−1^, which are assigned to the bending motion of *T*—O—*T* bonds in tetrahedra with *T* = Si, Al. The most intense band was deconvoluted into two contributions, at 487 and 468 cm^−1^, respectively (inset in Fig. S4). Other bending signals are found at ∼340 and ∼570 cm^−1^. A low-intensity signal was observed at ∼1040 cm^−1^, corresponding to the asymmetric stretching vibration of the Si—O bonds. In the low-wavenumber region, a Raman band at ∼130 cm^−1^ was assigned to the lattice modes within the tetrahedra of the zeolites (Knops-Gerrits *et al.*, 1997 ▸; Wopenka *et al.*, 1998 ▸). Within the sample, minor mineral phases were detected, in addition to the erionite contribution, as confirmed by the XRPD analysis. Colourless crystals of quartz and feldspars are mixed with the fibres. The Raman spectra of quartz and albite are shown in Fig. S5, detailing the main Raman features. Micrometric crystals with orange to brownish colour have also been found mixed with the fibres, showing the presence of iron-containing compounds (Fig. S6). They consist mainly of hematite, while the presence of goethite and magnetite is rare.

### 3.3. XRPD

The quantitative phase analysis (QPA) performed using the Rietveld method shows that the Tuzköy‘s tuff contains erionite 42.64 (4) wt%, clinoptilolite 24.8 (4), quartz 13.0 (1) wt%, sanidine 7.59 (4) wt%, hornblende 5.28 (4) wt%, albite 5.05 (2) wt% and mica 1.63 (1) wt%. The agreement indices of the Rietveld quantitative phase analysis are *R*_exp_ = 3.133, *R*_wp_ = 11.780%, *R*_p_ = 8.425%. The graphical output of the Rietveld refinement is shown in Fig. S7.

### 3.4. SEM

**Figure 2:**
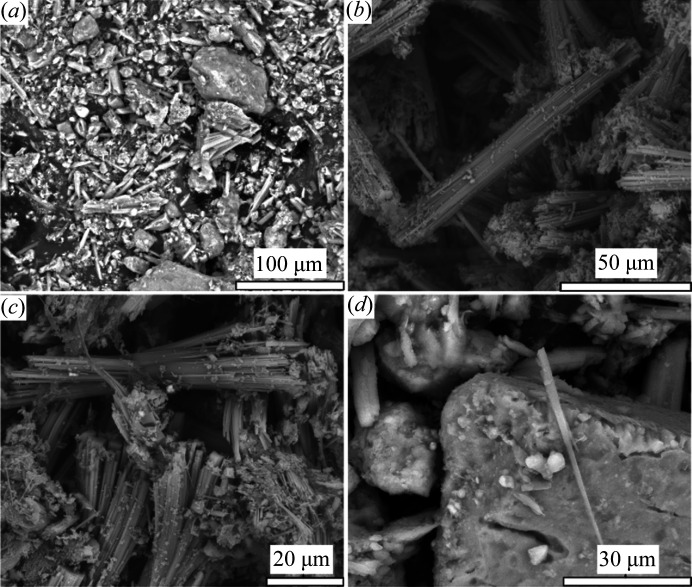
SEM pictures of Tuzkoy’s tuff and erionite fibres. (*a*) General overview of the sample (tuff). Erionite fibres are scarce in the matrix. (*b*) and (*c*) Representative high-resolution SEM-FEG images of fibre bundles. Erionite fibrils are grouped in tabular bundles with variable widths (*W*: between 2 and 11 µm) and an average length of 31 µm (*L*: between 15 and 47 µm). (*d*) Single fibril of erionite found in the sample.

A gallery of SEM images collected is reported in Fig. 2 ▸. The Tuzköy tuff is mainly composed of fibrous erionite crystals grouped in stocky bundles [Figs. 2 ▸(*a*)–2 ▸(*c*)] with variable *W* (between 2 and 11 µm) and *L* (between 15 and 47 µm). These bundles have a great tendency to slit into very fine fibrils [Fig. 2 ▸(*d*)]. The individual fibrils are dispersed into the matrix [Fig. 2 ▸(*a*)]. The morphology of the erionite fibres is prismatic or acicular and generally longer than 5.0 µm. They have a width ranging from 0.16 to 4.2 µm (Table S1 of the supporting information). Summary statistics of erionite fibre geometry (over 110 fibres) are reported in Table S1. All of the observed fibres have a *L*/*W* ratio >3:1. The X-EDS spectra were acquired on the bundles and single fibres (Fig. S8).

In agreement with the literature data, X-EDS data revealed the occurrence of Ca, Mg and K as extra-framework ions [Fig. S8(*c*)]. Also, a small amount of Fe content was detected [Fig. S8(*d*)]. However, high-resolution FEG-SEM images highlighted the presence of nanoparticles (with variable sizes) located at the surface of the bundle (Fig. S8b). It has already been shown that the presence of Fe in the erionite chemical analyses can be attributed to these impurity phases (Gualtieri *et al.*, 2016 ▸; Cametti *et al.*, 2013 ▸).

### 3.5. TEM

**Figure 3:**
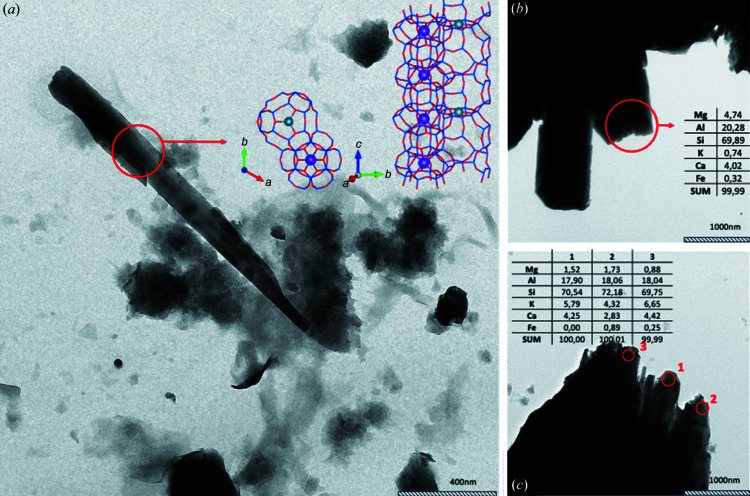
TEM image of erionite fibre bundles. (*a*) Relatively thin erionite fibre with a thickness of about 150 nm, from which a rather complete 3DED dataset was collected. *Ab initio* structure solution performed on this dataset allowed us to identify the erionite framework, which is overlapped in the figure. (*b*) and (*c*) Typical fringed appearance of large erionite bundles. Chemical analysis (EDX) performed on the red spots confirm the chemistry is in line with the erionite (data given as a percentage).

The TEM investigation was primarily performed to assess whether erionite from Tuzkoy presents offretit-disordered sequences. Fig. 3 ▸ shows typical bundles of erionite fibrils. TEM images confirmed that the variability of bundle dimensions is in line with the SEM study. As already described by Matassa *et al.* (2015 ▸), erionite tips often display a fringed appearance [Figs. 3 ▸(*b*) and 3 ▸(*c*)]. This evidence confirms that what is usually considered a fibre is a bundle of fibrils with a thickness of only a few tens of nanometres.

Several energy-dispersive X-ray (EDX) spectra coupled with 3DED were collected along thin erionite bundles or at the very tip of larger bundles. Representative chemical analyses are reported in Figs. 3 ▸(*b*) and 3 ▸(*c*). The red circles indicate the areas from which the chemical analyses were performed. The ribbon-like bundles revealed [in agreement with Ballirano *et al.* (2015 ▸)] the occurrence of Mg, Ca, K, Al and Si and, in some cases, a significant amount of Fe. As demonstrated by Gualtieri *et al.* (2018 ▸), Fe is not part of the erionite crystal structure but is associated with impurities that are mostly concentrated on the surface of the fibres. Cross-fibre EDS profiles show no significant change in chemical composition, ruling out the occurrence of clear polytypic sequences connected with chemical variations.

Although a relatively high Mg content was found, 3DED confirmed that all the specimens were characterized by lattice parameters consistent with erionite, and not with offretite. Although 3DED data did not prove to be as sensitive as synchrotron nano-diffraction for the extra-framework content, they were sufficient to solve the typical zeolite framework of erionite *ab initio* and to locate the K ion in the cancrinite cage, enforcing the evidence that offretite was not present.

### 3.6. Electron probe microanalysis

The results of the electron probe microanalyses (EPMA) expressed as weight percentages with standard deviations are reported in Table S2. The erionite chemical formula was calculated after renormalization of the chemical analyses, hypothesizing a water content of 18 wt% (corresponding to ∼30 water molecules per formula unit). The resulting chemical formula (K_2.63_Ca_1.57_Mg_0.76_Na_0.13_Ba_0.01_)[Si_28.62_Al_7.35_]O_72_·28.3H_2_O was obtained from the average of the chemical analyses of each sample passing the balance error (E%) (Passaglia, 1970 ▸).

### 3.7. SCXRD

**Figure 4:**
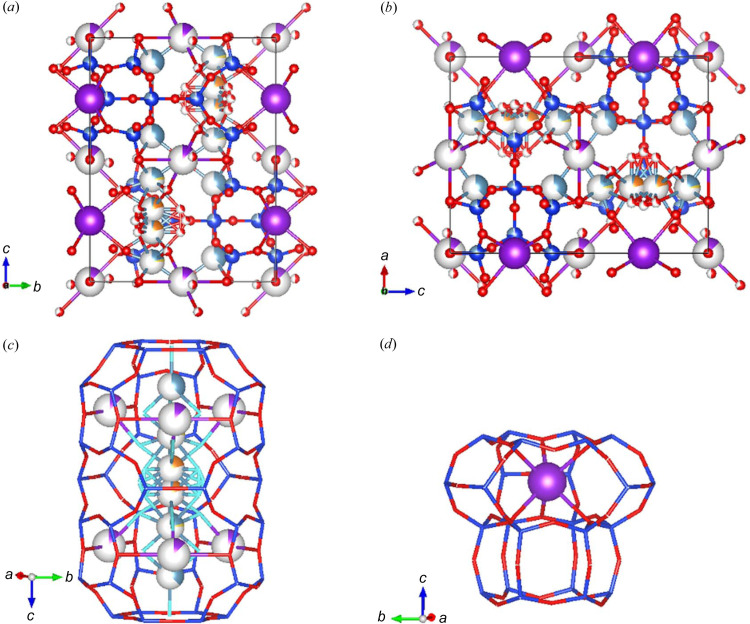
Crystal structure of erionite from Tuzköy (Turkey). (*a*) View of the crystal packing along the *a* axis. (*b*) View of the crystal packing along the *b* axis. (*c*) Detail of the erionite cavity and its extra-framework content. (*d*) Detail of the cancrinite cage hosting the K atom. Legend: electric blue balls = Si, red balls = O atoms (also H_2_O molecules), ice blue balls = Ca, yellow balls = Na, orange balls = Mg, purple balls = K. The plots were created using the *VESTA* software (Momma & Izumi, 2011 ▸).

The X-ray structural parameters of the erionite from Tuzköy collected at ID11 (ESRF) are reported in Table 1 ▸ and Figs. 4 ▸(*a*) and 4 ▸(*b*). It crystallizes in the hexagonal system, and systematic absences were consistent with the space group *P*6_3_/*mmc*. The unit-cell parameters *a* = *b* = 13.2708 (1) Å, *c* = 15.0958 (1) Å and *V* = 2302.40 (4) Å^3^ are slightly smaller than the values in the models previously proposed by Alberti *et al.* (1997 ▸), Gualtieri *et al.* (1998 ▸), Ballirano *et al.* (2009 ▸) and Cametti *et al.* (2013 ▸), showing that the ‘individual’ information derived from SCXRD data is close to the literature bulk information based on powder data (*i.e.* the *c*/*a* ratios of the model described below are identical within 1σ).

The refinement converged at *R*[*F* ^2^ > 2σ(*F* ^2^)] = 0.038, *wR*(*F* ^2^) = 0.114, *S* = 1.09. The analysed fibre is a fibril with a section whose diameter is about 200–300 nm isolated from larger ‘bundles’ of fibres.

The chemical partition after the final refinement was (K_2.64_Ca_2.61_Mg_0.60_Na_0.12_)[Si_28.53_Al_7.38_]O_100.80_ (omitting the contribution of the hydrogen atoms), in line with the calculated chemical analysis (K_2.63_Ca_1.57_Mg_0.76_Na_0.13_Ba_0.01_)[Si_28.62_Al_7.35_]O_72_·28.3H_2_O, except for the undetected traces of Ba and a higher content of Ca. Fractional coordinates, site partition (s.p.) atomic displacement parameters and site symmetry (s.s.) are reported in Table S4, and relevant bond distances are supplied in Table 2 ▸.

### 3.8. Framework

Two crystallographically independent tetrahedral sites were refined: *T*1 and *T*2, respectively, occupied by Al1 and Si1 (for *T*1), and Al2 and Si2 (for *T*2). These two sites, sitting on the positions *x*/*a* = 0.23398 (4), *y*/*b* = 0.99978 (3) and *z*/*c* = 0.10458 (3); and *x*/*a* = 0.33174 (4), *y*/*b* = 0.90620 (5) and *z*/*c* = 0.2500, build the D6R and S6R cages, respectively. Al1 and Si1, as well as Al2 and Si2, share the same site and, as such, their anisotropic thermal factors and occupancies have been constrained during the refinement. Free variables linked to the site-occupancy factors of Al and Si over the two crystallographic sites were refined with the restraint that the total percentage of each species has to satisfy the chemical analysis results (the target values), according to which Si is the dominant chemical species (79.57%) while Al corresponds to the remaining 20.43% of the total population of the two sites.

The mean bond distances 〈*T*1—O〉 = 1.637 Å and 〈*T*2—O〉 = 1.630 Å indicate a very small difference between the mean [T—O] distances of the two tetrahedral sites (〈*T*1—O〉 − 〈*T*2—O〉 is only 0.007 Å). Application of the Jones (1968 ▸) determinative curves indicates a small preference of Al for *T*2 (s.p. = 0.34) compared with *T*1 (0.14), in excellent agreement with both the chemical data and the refined site partition.

Individual *T*—O—*T* angles show no deviation from the values described by Alberti *et al.* (1997 ▸), Gualtieri *et al.* (1998 ▸) and Ballirano *et al.* (2009 ▸).

### 3.9. Extra-framework

The positions of the extra-framework atoms were identified by careful inspection of the electron-density map calculated by Fourier difference synthesis. The strong disorder of the extra-framework atoms and the presence of nearly isoelectronic cations reduced the discrimination power of the agreement factor *R*_F_; the criterion based only on the minimization of *R*_F_, if used stand-alone, was not sufficient for the correct assignment of the atomic species. To guide the structure completion process and correctly label the extra-framework atoms in the final refinement steps, the use of prior information on the chemical composition and the expected coordination of the cations was extremely helpful. The species were assigned based on the coordination and the distances from the surrounding water molecules of the candidate cations, in agreement with the chemical characterization results, by choosing the association that also minimized the *R*_F_ of the refinement to 3.8%. The extra-framework content was found to be the following:

The cancrinite cage hosts the K atom (*i.e.* K1) as shown in Fig. 4 ▸(*d*). This site is fully occupied, in line with the chemical characterization. As reported in the work by Gualtieri *et al.* (1998 ▸), this site may be fully occupied, as in the case of Lady Hill and Shourdo erionites, or only partially occupied (down to 85%) as in the case of the erionites found in Tunguska. The K1 atom is 12-fold coordinated with six O2 atoms (2.923 Å) and six O3 atoms (3.376 Å). The K1—O3 distance is longer than the K1—O2 distance. This difference is well reported by Cametti *et al.* (2013 ▸) for the woolly erionite-Na from Oregon. Another K site, K2, in position *x*/*a* = 0.5, *y*/*b* = 1 and *z*/*c* = 0, has been found with a site occupancy of 10%. K2 is coordinated to two O4 atoms (3.119 Å), four O1 atoms (3.276 Å), two oxygens OW3 (2.65 Å) and two oxygens OW1 (3.3192 Å); with the labels OW*j*, *j* = 1,…6, we refer to the oxygen atoms of the water molecules (due to the great disorder of the solvent, the hydrogens atoms of the water molecules were not positioned).

Several cation positions have been found in the erionite cage at different *z*/*c* heights as shown in Fig. 4 ▸(*c*).

The refinement indicated that the extra-framework cations are located at three Ca1, Ca2 and Ca3 sites, with the Ca2 and Ca3 sites partially substituted by Na and by all available Mg, respectively. We have also identified six water molecule sites.

The refined Ca population in erionite from Tuzköy shows a higher value than that reported in the chemical analysis. As in the work by Alberti *et al.* (1996 ▸), this difference can be explained by cation migration during the analysis or by the intrinsic difference of a bulk versus a single fibre/crystal analytical technique.

The Ca1 site is partially occupied and sits, along the axis of the erionite cage, at *z*/*c* = 0.4016 (14). The *z*/*c* coordinate is slightly displaced from the threefold axis. The Ca2 site is shared with the Na2 atom. Owing to some instabilities during the refinement, the Na2 occupancy was constrained to respect the same partition as derived from the chemical analysis, while the occupancy of the Ca2 was initially set free to be refined and then, once a refined value was obtained, it was fixed and some additional refinement cycles were carried out. Both Ca2 and Na2 have been constrained to the same anisotropic thermal factors.

The last site is Ca3, which is shared with the Mg3 atom. As already observed by others (Ballirano *et al.*, 2009 ▸), some difficulties have been encountered when modelling the occupancies of this site. This site is partially occupied at 21% (value originally obtained by refining the occupancy under the hypothesis that only Ca3 was present). The total content of Mg is in line with other erionites [see the woolly sample from Durkee, Oregon in the work by Cametti *et al.* (2013 ▸)]. Higher values can be used to discriminate between erionite and offretite. To rule out the presence of the latter, we performed a further crystallographic test, which is discussed in the following section.

**Figure 5:**
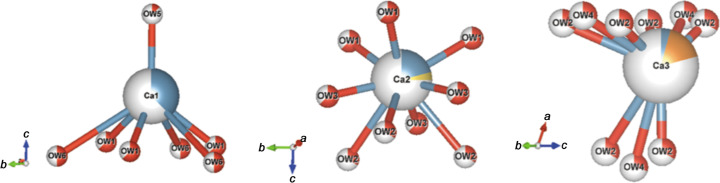
Ca site coordination (below 3 Å) along the [110] direction. (*a*) Ca1 coordinated to three OW1, three OW6 and one OW5. (*b*) Ca2 (partially occupied by Na2) coordinated to nine OW*j* atoms (three OW3, three OW1 and three OW2). (*c*) Ca3 is coordinated to six OW2 and three OW4. Legend: red = O atoms representing H_2_O molecules, ice blue = Ca, yellow = Na, orange = Mg. The plots were created using the *VESTA* software (Momma & Izumi, 2011 ▸).

According to the bond analysis (below 3.3 Å; Table 2 ▸) and Fig. 5 ▸, Ca1 is surrounded by three OW1 at a distance of 2.33 Å, three OW6 at a distance of 3.13 Å and one OW5 at a distance of 2.29 Å. Ca2 (site shared with Na2) is connected instead to nine OW*j* atoms (three OW3 at 2.06 Å, three OW1 at 2.26 Å and three OW2 at 2.54 Å, respectively). Ca3 (site shared with Mg) is coordinated to nine OW*j* atoms, *i.e.* six OW2 (three of them at a distance of 1.77 Å and the rest at a distance of 2.25 Å) and three OW4 at distances of 2.20 Å. Six oxygen sites assigned to H_2_O were found using difference Fourier maps; these are all located in the erionite cage as shown in Fig. S9. Concerning the oxygen positions of the water molecules, some differences are found compared with the model described by Cametti *et al.* (2013 ▸), Ballirano *et al.* (2009 ▸) and Gualtieri *et al.* (1998 ▸), but this is not surprising because the content and position of H_2_O molecules in erionite samples are extremely variable. All water site occupancies have been refined and are displayed in Fig. S9. The ISOR weak restraint for all the oxygen atoms of the water molecules has been applied to allow approximately isotropic refinement. The *PLATON* alert (PLAT260_ALERT_2_B) concerning OW1 and OW6 may be attributed to solvent disorder in the erionite channels.

## 4. Discussion

This study reports the full crystal-chemical and structural characterization of a mesotheliomagenic erionite fibre from Tuzköy (Turkey). We have used the term ‘killer’ fibre because it is universally shared that fibrous erionite is directly responsible for fatal lung malignancies (namely MM) in the population living in the Cappadocian area of Turkey (Carbone *et al.*, 2011 ▸). The potency of fibrous erionite in inducing MM in rats has been observed by Wagner *et al.* (1985 ▸). Carthew *et al.* (1992 ▸) reported that erionite has 300–800 times more MM potency than chrysotile and 100–500 times more such potency than crocidolite when given through intrapleural routes in animals. These data are confirmed by other animal studies showing that erionite is 200 times more tumorigenic than crocidolite (Hill *et al.*, 1990 ▸) and 500–800 times more tumorigenic than chrysotile (Coffin *et al.*, 1992 ▸). In the Cappadocian region, exposure to fibrous erionite caused an MM epidemic that was unprecedented in history in the villages of Karain, Sarihidir and Tuzköy as a result of the inhabitants building their homes from erionite-rich pyroclastic rocks (Carbone *et al.*, 2011 ▸).

Although genetic susceptibility has been invoked to explain the aetiology of MM (see below) and especially the high potency of erionite in inducing MM in humans (Carbone & Yang, 2012 ▸), the peculiar crystal physicochemical properties (surface iron, cation exchange, biodurability) of fibrous erionite and their interplay with extrinsic factors explain its carcinogenic potential. For this reason, the determination of the crystal chemistry of the erionite fibre from Tuzköy (Turkey) allowed us to reconstruct a detailed physical profile of this ‘killer’ substance.

### 4.1. The offretite dilemma

Erionite is often associated with the ‘sister’ zeolite offretite [OFF] with the ideal formula K_2_Ca_2_Mg_2_[Al_10_Si_26_O_72_]·32H_2_O, hexagonal with unit-cell parameters of approximately *a* = 13.29, *c* = 7.58 Å and the space group *P* 6 *m*2 (Passaglia *et al.*, 1998 ▸). Because erionite–offretite epitaxial growths and disordered erionite–offretite stacking sequences are common (Passaglia *et al.*, 1998 ▸), especially in Mg-rich samples, there is a need for precise identification of the erionite fibre from Tuzköy to rule out an offretite or an erionite–offretite sequence. This point is very important due to the implication of erionite in causing MM. If it is discovered that the studied crystal is offretite or a mix erionite–offretite instead, there would be resounding implications at health, regulatory and legal levels. First of all, offretite should be explicitly included in the list of IARC carcinogens together with erionite while, at present, it is only suspected to have toxic potential similar to that of erionite (Mattioli *et al.*, 2018 ▸).

Although it is very difficult to distinguish erionite from offretite, because they have similar cation content and crystal structures, the results of our study unequivocally rule out the possibility that erionite from Tuzköy is actually offretite.

Our attention was first focused on the Mg content. The Mg values (Mg_0.60_ from the structure refinement and Mg_0.76_ from the EPMA, respectively) indicate that we are in the composition range of erionite and not in that of offretite, which shows a limited variation and Ca/Mg ratio very close to 1.0 (Passaglia *et al.*, 1998 ▸).

**Figure 6:**
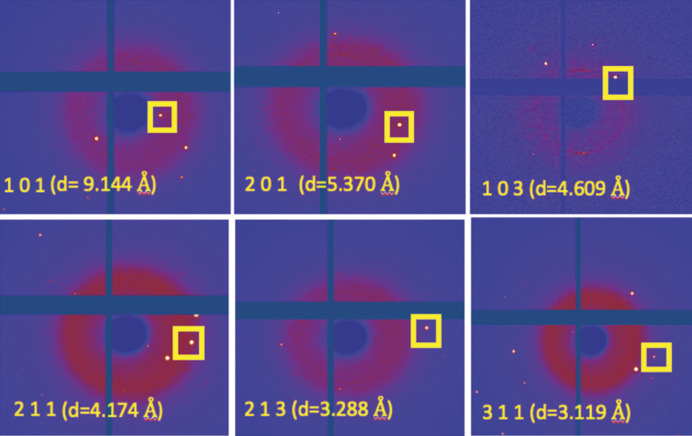
Assessing the presence of the erionite characteristic reflections (101), (201), (211), (213) and (311) in the raw images. The cross in the image corresponds to a module gap present in the detector Eiger2 4M CdTe. The additional reflection (103), to be ascribed only to erionite, is shown.

From a crystallographic standpoint, the metric relationship existing between the cell parameters of the two structures suggests that most of the Bragg peaks of the two zeolites coincide exactly in their diffraction patterns (Kerr *et al.*, 1970 ▸; Bennett & Grose, 1978 ▸). In more detail, the *l* = 2*m* (*m* ≠ 0) *hkl* reflections of the erionite perfectly overlap the (*hkm*) reflections of the offretite, as well as the (*hk*0) of erionite with the (*hk*0) of the offretite. There are, however, a few relatively strong diffraction lines due to the unit-cell doubling [namely the (101), (201), (211), (213), (311) reflections (Passaglia *et al.*, 1998 ▸)] that only belong to erionite. The appearance of these reflections is considered a further validation for the presence of erionite. Fig. 6 ▸ shows the assessment of these (*hkl*) reflections in the raw images collected during the single-crystal experiment.

Nevertheless, if we consider low-θ (high *d*-spacing) indices, there is another reflection, namely (103), that the work of Passaglia *et al.* (1998 ▸) did not include in the list. A plausible reason why Passaglia *et al.* (1998 ▸) do not mention the (103) reflection may be because the results were obtained from powder diffraction and so this reflection tends to be overlapped with the (202). This additional characteristic reflection may still improve the ‘diagnostic’ for testing the effective presence of the erionite with sufficiently high-resolution powder data.

**Figure 7:**
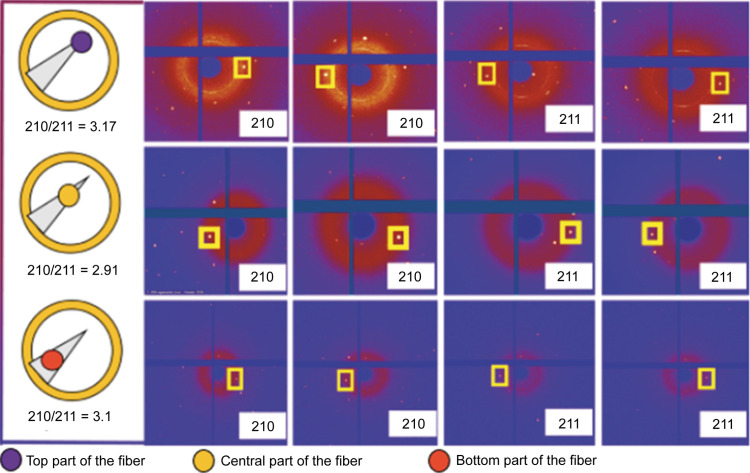
Evaluation of the ratio for the diffraction spots intensities 210/211 to rule out the presence of offretite as described by Passaglia *et al.* (1998 ▸). The left section represents the point at which the measure was performed. Statistical errors on the intensity ratios are 0.02.

The test described above, however, does not rule out the presence of offretite; thus a method considering the intensity ratio of Bragg peaks containing only the erionite scattering contribution to those containing both the erionite and the offretite contributions could be used to determine the offretite content in erionite. This method described by Passaglia *et al.*, (1998 ▸) has been applied by normalizing the intensity of the 210 peak (present in both erionite and offretite) to clearly show the change in the 210/211 [only observable in erionite, owing to (211)]. The same method has been applied to our single-crystal study (Fig. 7 ▸). In our case, the data used for this test were collected at different heights of the investigated fibre which is prismatic and does not have a constant volume exposed to the X-rays. This means that, for different measures in height, the scattering intensity must not be the same. If some intercalation of offretite was present, the ratio between 210 and 211 integrated intensities would change. For this test, this value remains substantially constant and this rules out the possibility of offretite stackings.

### 4.2. Classification of the erionite fibre

Given that the Tuzköy fibre is not offretite, this species should be classified within the erionite family. The chemical formula calculated from the refinement using SCXRD is (K_2.63_Ca_2.62_Mg_0.60_Na_0.13_)[Si_28.56_Al_7.44_]O_72_·28.80H_2_O and indicates an erionite-K/erionite-Ca term. This is different from the mean chemical formula obtained from the EPMA that is (K_2.63_Ca_1.57_Mg_0.76_Na_0.13_Ba_0.01_)[Si_28.62_Al_7.35_]O_72_·28.30H_2_O and points to an erionite-K term. The two formulae differ mainly for the Ca content and this can be due to several reasons: (i) EPMA data were obtained by averaging various points sampled on several eventually different fibres; (ii) chemical characterization was performed using a bulk technique, whereas the crystallographic characterization was performed on a single fibre; (iii) erionite possesses intrinsic variability, as also demonstrated in the EDX spectra collected on different erionite fringes; (iv) chemical point analysis using sources of electrons can be problematic (Clark *et al.*, 1995 ▸) when the target is a thin mineral fibre or fibre bundle because there is a strong influence of the shape and thickness of these anisotropic particles on the detected signal (Valdrè *et al.*, 2018 ▸). Also for EDS-based determinations, especially for long analysis times, possible beam damage of the zeolite fibre and loss of low-*Z* elements, particularly Na, may be expected (Dogan, 2012 ▸).

The chemical variability of the Cappadocian erionites is confirmed by point chemical analyses determined by Dogan (2012 ▸), indicating both erionite-K and erionite-Ca terms with a prevalence of the erionite-K for the TEM-EDS results independently verified by EPMA. Dogan (2012 ▸) proposed the mean formula (K_3.09_Ca_1.57_Mg_0.55_Na_0.26_)[Si_28.70_Al_6.61_Fe_0.60_]O_72_ (water molecules omitted) in agreement with our EPMA-determined formula.

### 4.3. Environmental and health implications of the study

The crystal structure of the mesotheliomagenic erionite fibre from Tuzköy will aid the understanding of the biochemical mechanisms that cause adverse effects *in vivo* and lead to the onset of MM. The profile of this erionite ‘killer’ fibre rules out the role of the other ‘suspects’: offretite or erionite–offretite disordered sequences.

Tuzköy fibres are longer than 5.0 µm and display widths in the range 0.16 to 4.2 µm (Table S1). Geometrically, these erionite fibres cannot negotiate the aperture (3–10 µm wide) of the pleural diaphragmatic stomata and can undergo phagocytosis attempts by phagocytic cells leading to inflammation in the pleural space. Because erionite fibres are biodurable and phagocytosis is inefficient, the inflammation activity is chronic and causes damage to the DNA of the adjacent mesothelial cells, initiating the sequence of adverse effects leading to the onset of MM.

The Tuzköy erionite fibre does not host iron in the structure, but micrometric iron-rich particles of hematite and subordinate goethite and magnetite can be found at the surface of the fibres. These iron-rich impurities may be responsible to produce ROS (Gualtieri *et al.*, 2016 ▸; Gualtieri, 2018 ▸) and can dissolve during partial phagocytosis when the erionite fibres are engulfed in the acidic phago-lysosome sacks, leaving a residue of iron atoms at specific catalytic sites anchored to the surface windows of the six-membered rings of erionite. The newly formed iron species form cyto/geno-toxic free radicals when they are in contact with H_2_O_2_ released during phagocytosis.

We have also seen that the Tuzköy fibre is rich in Ca^2+^ and K^+^ extra-framework cations. These cations can be exchanged in both extracellular and intracellular media (Di Giuseppe *et al.*, 2022 ▸). In lung lining fluid of the extracellular environment, the cation content is: K^+^ 6–29 m*M*, Na^+^ 82–132 m*M*, Ca^2+^ 4 m*M* while in the cytosol (intracellular environment) the cation content is K^+^ 139–150 m*M*, Na^+^ 12 m*M*, Ca^2+^ 2 × 10^−4^ m*M* (Lodish *et al.*, 1999 ▸; Innes *et al.*, 2021 ▸). Considering the cation-exchange properties of erionite [with the selectivity series Rb^+^ > Cs^+^ > K^+^ > Ba^2+^ > Sr^2+^ > Ca^2+^ > Na^+^ > Li^+^ (Sherry, 1979 ▸)], the Tuzköy fibre can adsorb and trap K^+^ in its micropores and release Ca^2+^ in both extracellular and intracellular environments. The minor amount of extracellular and intracellular Ca^2+^ can also be exchanged by extra-framework Na^+^ (and eventually Mg^2+^).

It is still unknown whether Ca^2+^ exchange interferes with calcium cross-talk in the cytosol. The latter is assumed to be one of the most important biochemical mechanisms controlling cell survival/proliferation. According to the model delivered by Carbone & Yang (2012 ▸), cells with extensive DNA damage caused by exposure to erionite undergo programmed death (apoptosis) and do not grow into malignancies due to the action of genes like BAP1. In the endoplasmic reticulum (ER), BAP1 protein binds, deubiquitylates and stabilizes type 3 inositol-1,4,5-tris­phosphate receptor (IP3R3), modulating Ca^2+^ release from the ER into the cytosol and mitochondria to promote apoptosis (Bononi *et al.*, 2017 ▸). Reduced levels of BAP1 in the genetically predisposed carriers of the mutated BAP1^±^ forms are responsible for the reduction both of IP3R3 levels and Ca^2+^ flux, preventing BAP1^±^ cells that accumulate DNA damage from executing apoptosis. A higher fraction of cells exposed to erionite survives genotoxic stress, resulting in a higher rate of cell transformation and proliferation and a higher probability of onset carcinogenesis. Ca^2+^ exchange induced by erionite can modify cytosol ion concentration and eventually alter the ER-mitochondria cross-talk (calcium ATPase pump) to restrain or interrupt the mitochondrial apoptotic pathways in the same way as the lack of BAP1 (substituted by the modified forms BAP1^±^) does. Hence, intracellular Ca^2+^ exchange may be a co-factor in determining the mesothelioma-genicity of erionite-like genetic susceptibility.

Finally, we note that the behaviour of erionite-K/erionite-Ca from Tuzköy should differ from that of other erionite species like erionite-Na from Jersey (Nevada, USA) (Gualtieri *et al.*, 2016 ▸) for which extracellular/intracellular K^+^ and Ca^2+^ exchange by Na^+^ should be more efficient.

More work is needed to understand the cation-exchange mechanisms of erionite *in vitro* and *in vivo* as pointed out by Pacella *et al.* (2021 ▸) who suggested that the biological effects hypothesized for the released cations (such as Mg^2+^ and Ca^2+^) may be investigated by comparing the *in vitro* toxicity of both pristine and modified samples after immersion in lung fluids.

## 5. Conclusions

This study reports the full crystal-chemical characterization of an erionite fibre from Tuzkoy (Cappadocia, Turkey). The state-of-the-art combined approach of n-SCXRD, TEM and µ-Raman techniques allowed us to determine that the erionite from Tuzkoy is not associated with offretite. These findings are relevant owing to the implication of erionite causing mesothelioma, and at regulatory levels and represent the onset for *in vivo* and *in vitro* studies to understand its toxicity. The exact determination of the extra-framework content, and more precisely K and Ca, is crucial to determine the mechanism by which it may be exchanged in extracellular and intracellular media.

Until now, most of the erionite crystal structures described in the literature were obtained from powder diffraction methods. These nanometric fibres are difficult to isolate and it is even more challenging to collect good datasets. Thanks to the new upgraded machine of the ESRF (Extra Brilliant Source) and new-generation hybrid photon counting detectors (Eiger2 4M CdTe), it is now possible to collect reliable and complete datasets of nanometre-sized crystals that allow a very detailed structural study of complicated systems such as this fibrous zeolite.

## Supplementary Material

Crystal structure: contains datablock(s) I. DOI: 10.1107/S2052252523003500/ti5026sup1.cif

Structure factors: contains datablock(s) I. DOI: 10.1107/S2052252523003500/ti5026sup2.hkl

Supporting figures and tables. DOI: 10.1107/S2052252523003500/ti5026sup3.pdf

CCDC reference: 2256957

## Figures and Tables

**Table 1:** Crystallographic data of the single-crystal structure refinements of erionite from Tuzköy Standard deviations are given in parentheses.

Crystal data	
Chemical formula	Al_7.44_Ca_2.62_K_2.63_Mg_0.60_Na_0.13_O_100.80_Si_28.56_
*M*_r_	2812.59
Crystal system, space group	Hexagonal, *P*6_3_/*mmc*
Temperature (K)	293
*a*, *c* (Å)	13.2708 (1), 15.0958 (1)
*V* (Å^3^)	2302.40 (4)
*Z*	1
Radiation type	Synchrotron, λ = 0.3257 Å
μ (mm^−1^)	0.12
Crystal size (mm)	0.00035 × 0.00054 × 0.02

Data collection	
Diffractometer	Id11 nanoscope
Absorption correction	SCALE3 ABSPACK
No. of measured, independent and observed [*I* > 2σ(*I*)] reflections	45831, 1357, 1322
*R*_int_	0.054
(sin θ/λ)_max_ (Å^−1^)	0.714

Refinement	
*R*[*F* ^2^ > 2σ(*F* ^2^)], *wR*(*F* ^2^), *S*	0.038, 0.114, 1.09
No. of reflections	1357
No. of parameters	109
No. of restraints	39
Δρ_max_, Δρ_min_ (e Å^−3^)	1.08, −0.46

**Table 2:** Relevant bond distances (Å) of erionite from Tuzköy (Turkey) (*T*1 = Si1, Al1, *T*2 = Si2, Al2)

*T*1—O4		1.6311 (7)	Ca1—OW1 ×3			2.33 (3)
*T*1—O1		1.6335 (13)	Ca1—OW5			2.29 (2)
*T*1—O3		1.6363 (7)	Ca1—OW6 ×3			3.13 (5)
*T*1—O2		1.6462 (8)	Ca1—Ca2			2.71 (3)
Mean		1.636775	Ca2—Ca3			1.86 (2)

*T*2—O6		1.6154 (6)	Ca2—OW3 ×3			2.06 (2)
*T*2—O5		1.6334 (9)	Ca2—OW1 ×3			2.26 (3)
*T*2—O1 ×2		1.6409 (13)	Ca2—OW2 ×3			2.54 (7)
Mean		1.6299				
			Ca3—OW2 ×3			1.77 (3)
			Ca3—OW4 ×3			2.20 (6)
K1—O2 ×6		2.9228 (19)	Ca3—OW2 ×3			2.25 (3)
K1—O3 ×6		3.376 (2)				
			
K2—OW1 ×2		3.3192 (8)				
K2—OW6 ×2		1.19 (4)				
K2—OW3 ×2		2.65 (3)				
K2—O4 ×2		3.119 (2)				
K2—O1 ×4		3.2758 (14)				
