# Supplementary material for: The crystal structure of the killer fibre erionite from Tuzköy (Cappadocia, Turkey)
Source: IUCrJ. 2023 May 18;10(Pt 4):397–410. doi: 10.1107/S2052252523003500 (PMC10324483; doi:10.1107/S2052252523003500)
Supplement: Supplementary file 3 [file m-10-00397-sup3.pdf]

IUCrJ

Volume 10 (2023)

Supporting information for article:

The crystal structure of the killer fibre erionite from Tuzköy (Cappadocia, Turkey)

Carlotta Giacobbe, Anna Moliterni, Dario Di Giuseppe, Daniele Malferrari, Jonathan P. Wright, Michele Mattioli, Simona Ranieri, Cinzia Giannini, Laura Fornasini, Enrico Mugnaioli, Paolo Ballirano and Alessandro F. Gualtieri

Supplementary information

Figure S1. Erionite fibre mounted on a Dual-Thickness MicroLoops LD™ of 50 µm aperture.

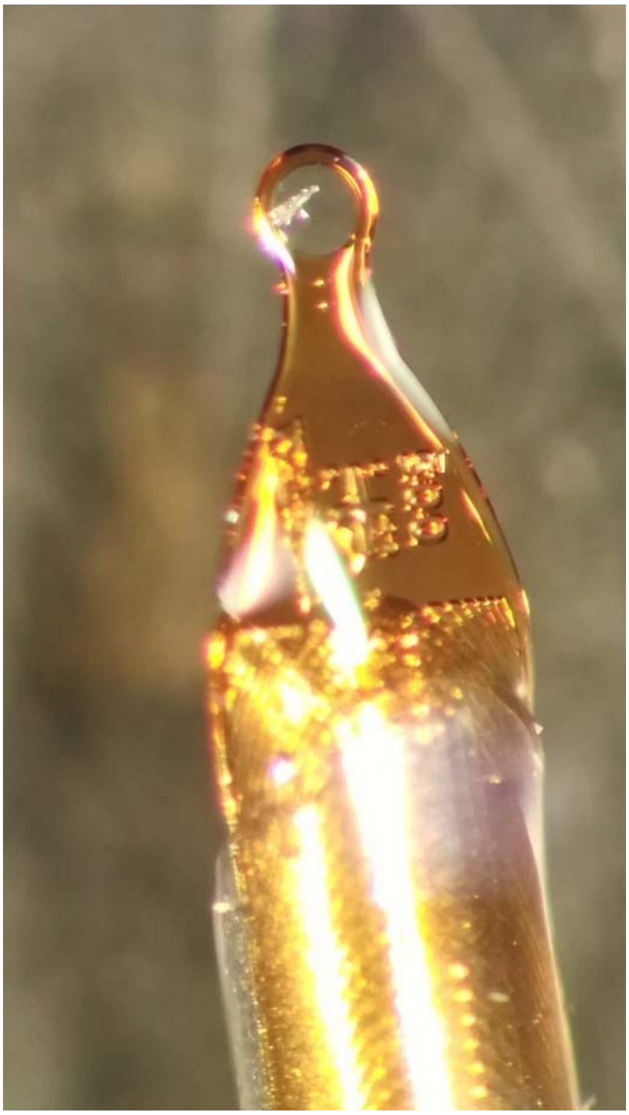

Figure S2 (a) Fit of the crystal size of the erionite fibre by scanning the motor "px" across the beam (x dimension of the fibre): resulting size is 351 nm. (b) Fit of the crystal size of the erionite fibre by scanning the motor "py" across the beam, after a rotation at 90 degrees on z (y dimension of the fibre): resulting size is 545 nm.

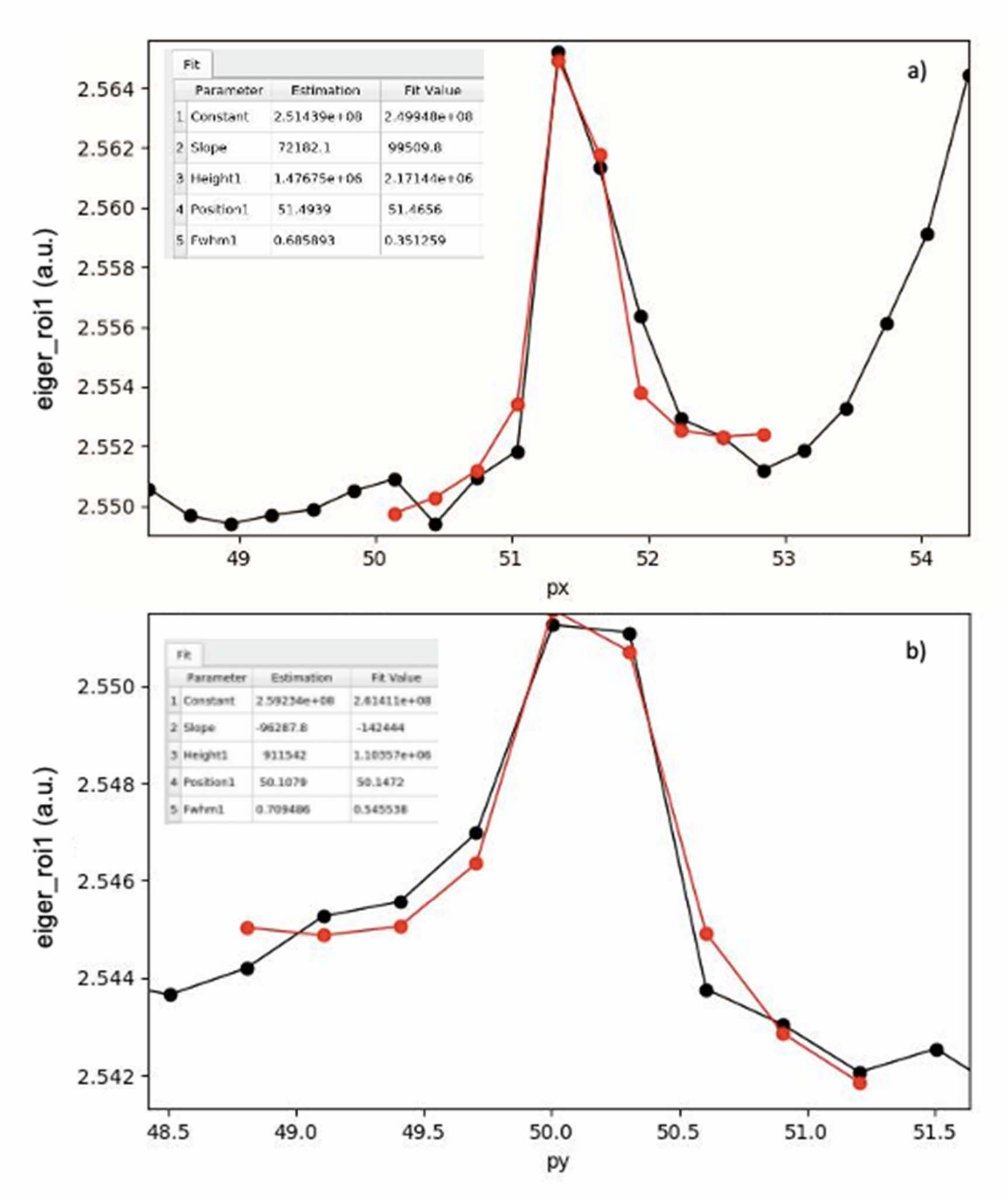

Figure S3. Thermogravimetric analysis (TG, top grey line), TG first derivative (DTG, top black line) and differential thermal analysis (DTA, bottom line) curves of Tuzkoy's tuff powder.

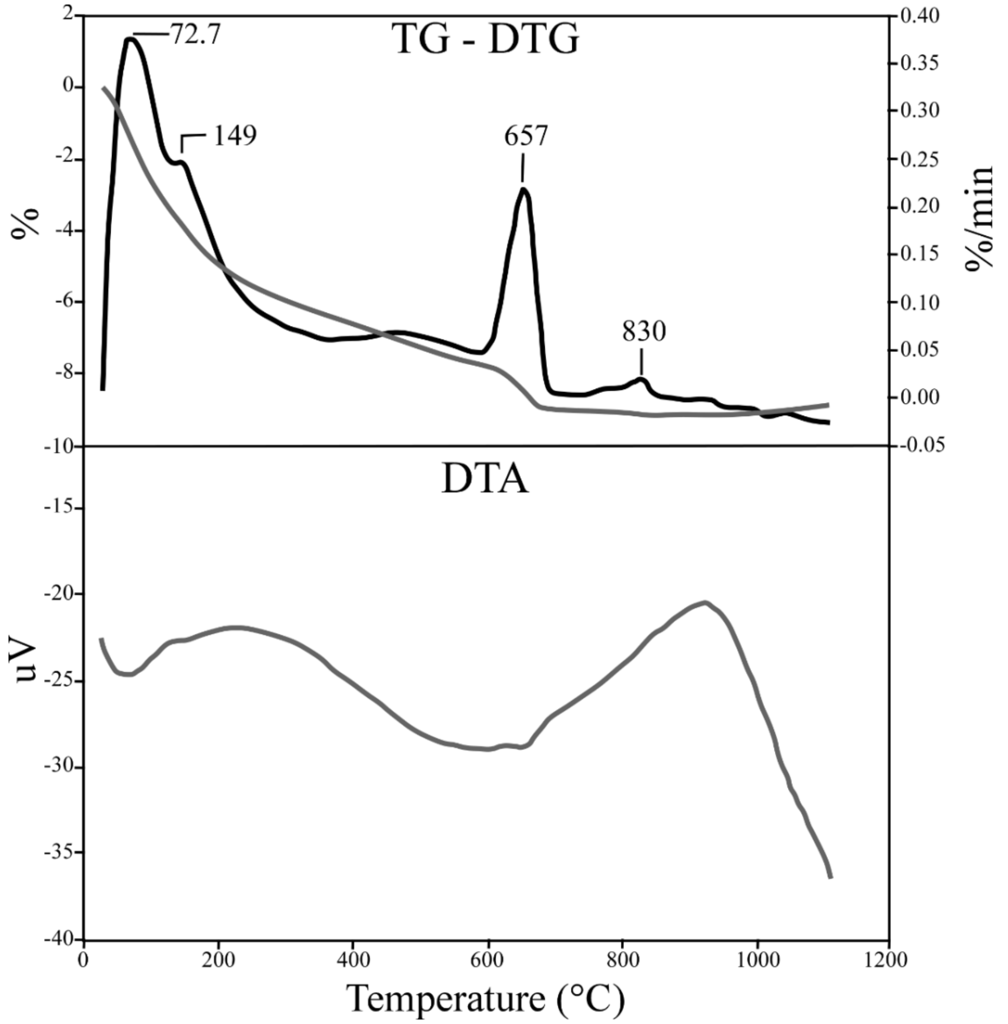

Figure S4. Raman spectrum of erionite from Tuzköy (Turkey) compared to Raman spectra of erionite from Jersey (Nevada, USA) and offretite from Saviore dell'Adamello, Brescia (Italy). In the insets the deconvolution of the Raman bands in the 400-600 cm^{-1} spectral region is shown.

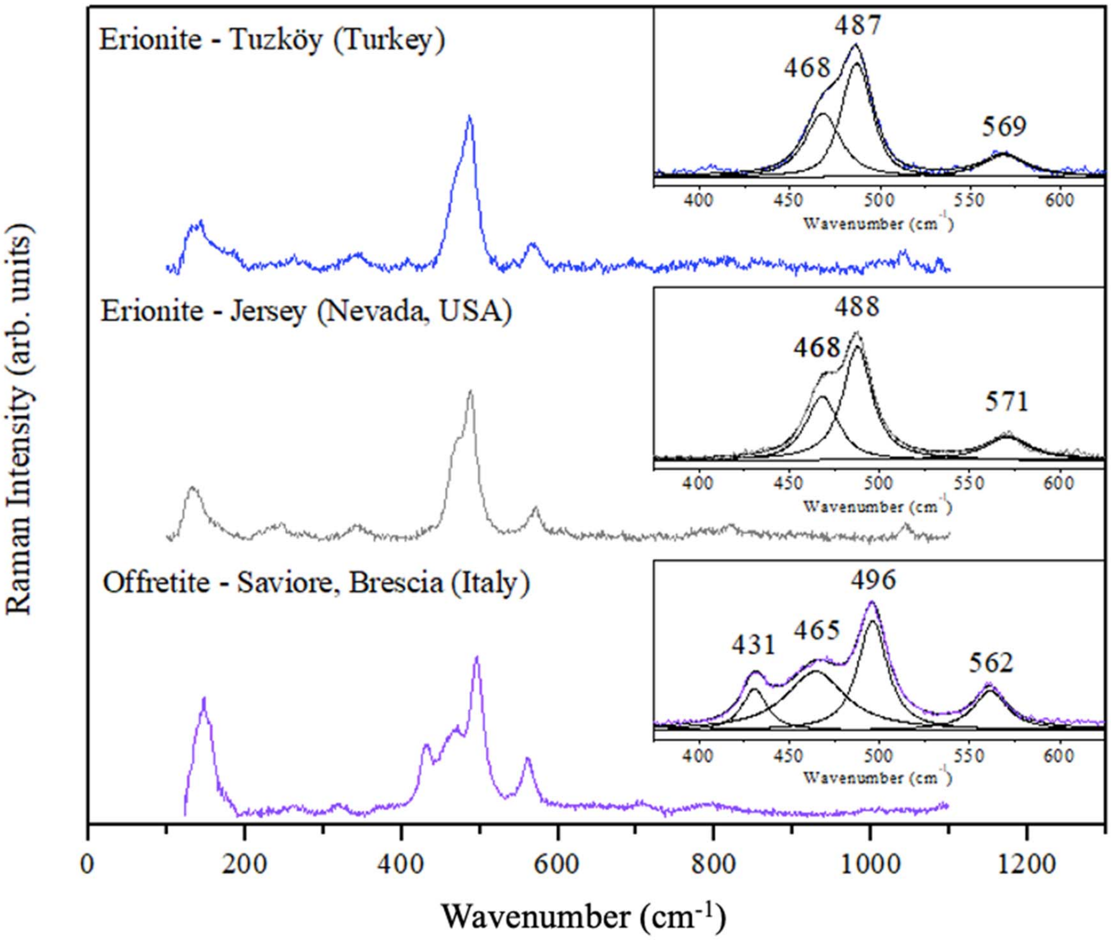

Figure S5. Raman spectra of quartz and albite crystals found in the sample.

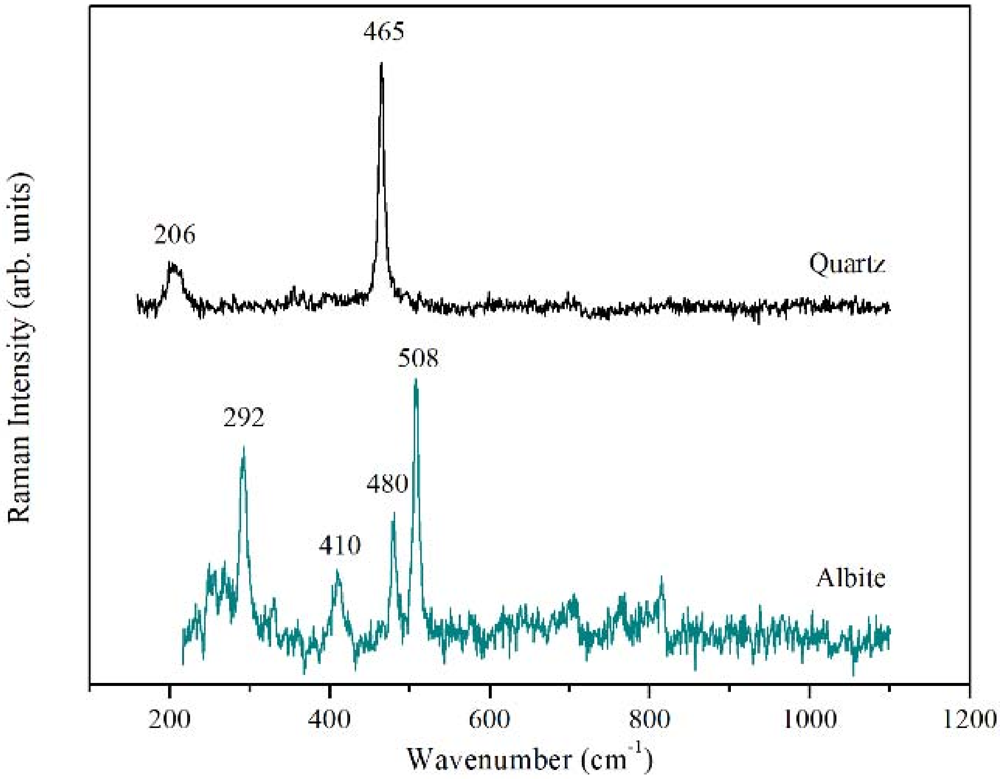

Figure S6. Raman spectra of iron-containing compounds found in the sample, showing hematite, goethite and magnetite signals. Traces of quartz in the goethite spectrum are identified by the peak at 465 cm^{-1}, marked with the * symbol.

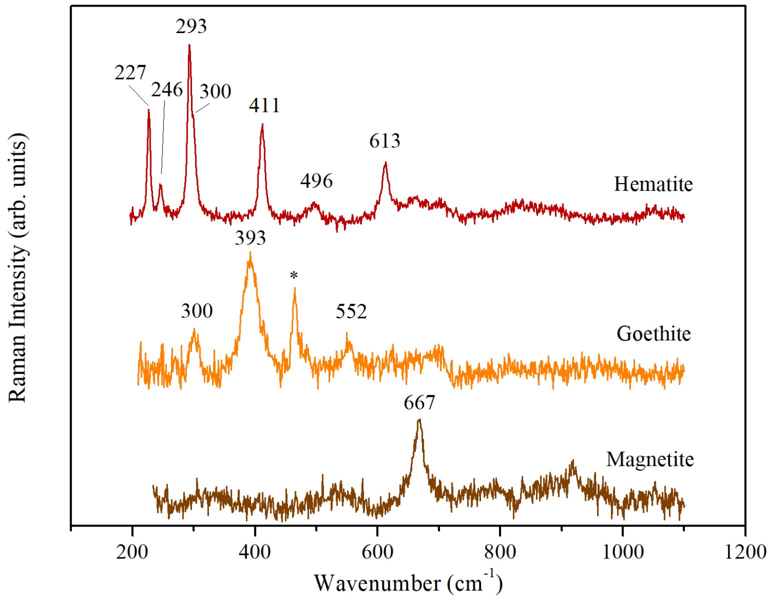

Figure S7 Rietveld refinement of the tuff pattern containing the erionite fibres. Blue line: experimental data; red line: calculated pattern; grey line: difference. Thickmark legend: blue= quartz, black= erionite, dark green= sanidine, fushia= albite, purple= clinoptilolite, green=horneblende, brown=mica.

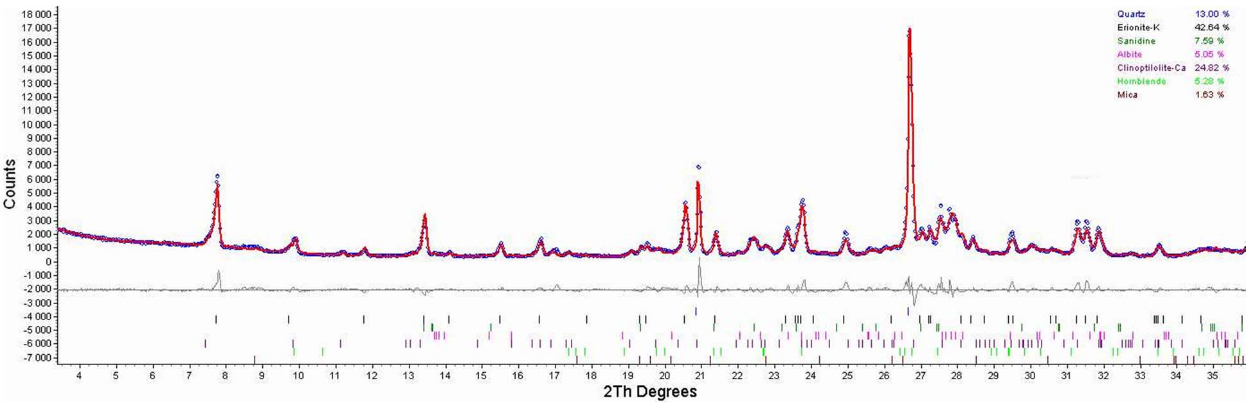

Table S1. Summary statistic of erionite fibres geometry. L (length); W (width); Min (minimum); Max (maximum); SD (standard deviation).* Percentile is a value below which a given percentage of values in a data set fall. Example: Given a group of observations, the 25th percentile is the value that is greater or equal to 25% of the observations, i.e. the 25% of fibres have L ≤ 11.0 µm, W≤ 0.54 µm and L/W ≤ 14.6.

		Percentiles*						
	Min	5th	25th	50th	75th	95th	Max	SD
L (μm)	5.03	6.19	11.0	17.1	24.9	45.1	55.9	11.7
W (μm)	0.16	0.29	0.54	0.81	1.15	1.77	4.20	0.57
L/W	6.66	9.51	14.6	21.9	30.4	41.7	74.8	11.6

Figure S8. SEM microphotographs of Tuzkoy's tuff and erionite fibres and X-EDS spectra. a) Single fibre of erionite found in the sample. b) High resolution FEGSEM images revealed the presence of micro-particles (black circle) resting at the bundles surface. c) Typical EDS spectrum recorded on mordenite surface free from the particles. No iron was found. (d) Representative EDS spectrum of the microparticles present in the fibres surface. A peak produced by iron is observed.

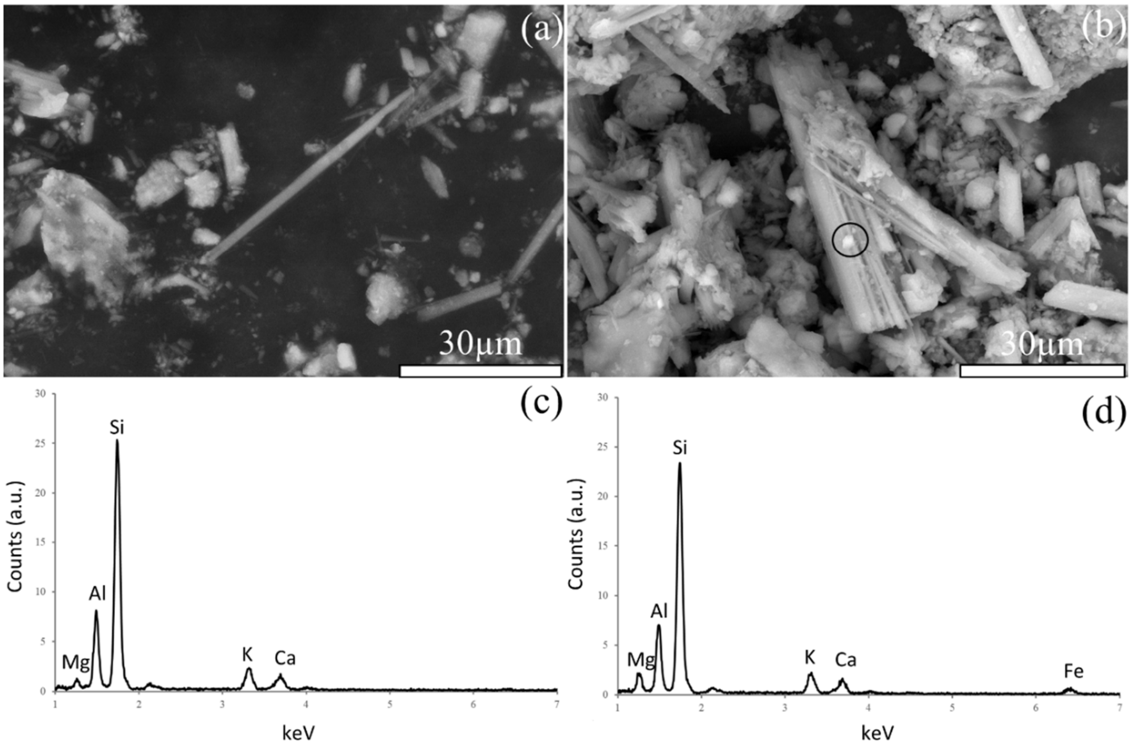

Table S2. Chemical compositions deriving from EMPA. Apfu on the basis of 72 oxygens H_2O fixed at 18.50 wt%

Wt%	Average
SiO_2	62.44
Al_2O_3	13.60
MgO	1.12
CaO	3.20

BaO	0.04
Na_2O	0.15
K_2O	4.49
Total	85.02
apfu	Average
Si	28.62
Al	7.35
Mg	0.76
Ca	1.57
Ba	0.01
Na	0.13
K	2.63
H_2O	28.30
E%	-0.97

Figure S9 H_2O sites found by Fourier map in the erionite cage.

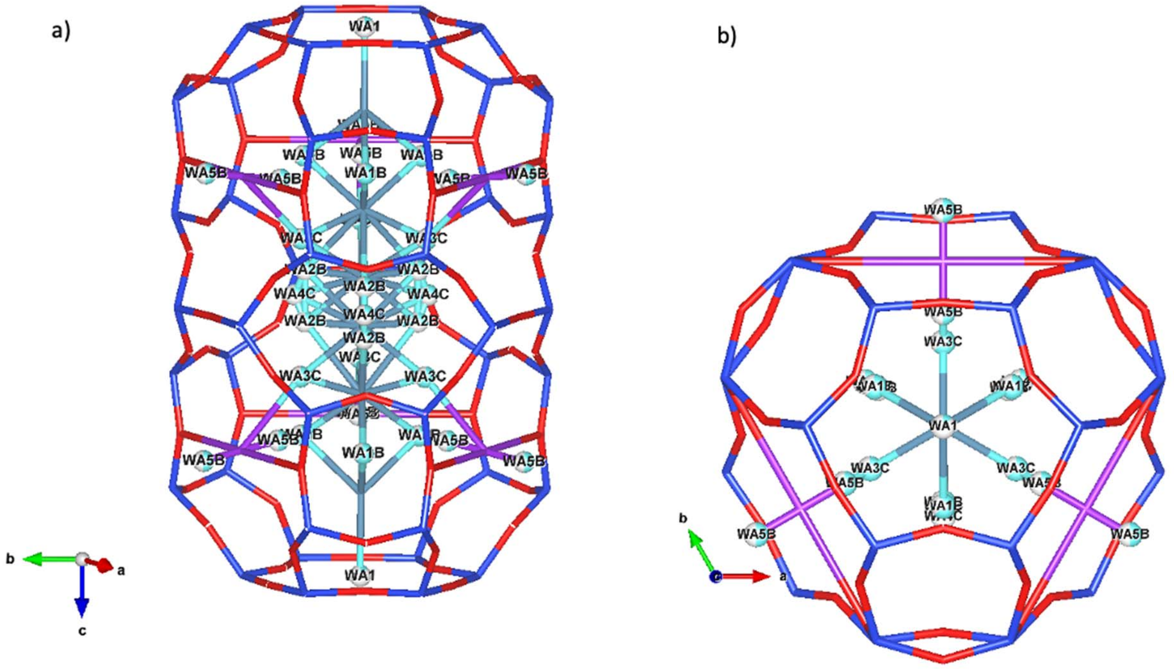

Table S3. Selected geometric parameters (Å, º)

Si1—O4	1.6311 (7)	K2—WA1B^{xi}	3.3192 (9)

Si1—O1	1.6334 (13)	K2—WA1B^{xii}	3.3192 (9)
Si1—O3	1.6363 (7)	Ca1—WA1	2.29 (2)
Si1—O2	1.6462 (8)	Ca1—WA1B^{xiii}	2.33 (3)
Si1—K1	3.8039 (4)	Ca1—WA1B^{xiv}	2.33 (3)
Si1—K2	3.8658 (4)	Ca1—WA1B^{xv}	2.33 (3)
Si2—O6	1.6154 (6)	Ca1—Ca2^{xiv}	2.72 (3)
Si2—O5	1.6334 (9)	Ca1—WA5B^{xvi}	3.13 (5)
Si2—O1	1.6410 (13)	Ca1—WA5B^{i}	3.13 (5)
Si2—O1^{i}	1.6411 (13)	Ca1—WA5B^{xvii}	3.13 (5)
K1—O2^{ii}	2.9228 (19)	Ca2—Ca3	1.86 (2)
K1—O2^{iii}	2.9228 (19)	Ca2—WA3C^{xviii}	2.06 (2)
K1—O2^{i}	2.9228 (19)	Ca2—WA3C	2.06 (2)
K1—O2^{iv}	2.9228 (19)	Ca2—WA3C^{xix}	2.06 (2)
K1—O2^{v}	2.9228 (19)	Ca2—WA1B	2.26 (3)
K1—O2	2.9228 (19)	Ca2—WA1B^{xix}	2.26 (3)
K1—O3^{iii}	3.376 (2)	Ca2—WA1B^{xviii}	2.26 (3)
K1—O3^{i}	3.376 (2)	Ca2—WA2B^{i}	2.54 (7)
K1—O3^{ii}	3.376 (2)	Ca2—WA2B^{vii}	2.54 (7)
K1—O3^{v}	3.376 (2)	Ca2—WA2B^{xi}	2.54 (7)
K1—O3^{iv}	3.376 (2)	Ca3—Ca3^{i}	1.36 (4)
K1—O3	3.376 (2)	Ca3—WA2B^{vii}	1.77 (3)
K2—WA5B^{vi}	1.19 (4)	Ca3—WA2B^{i}	1.77 (3)
K2—WA5B	1.19 (4)	Ca3—WA2B^{xi}	1.77 (3)
K2—WA3C^{vii}	2.65 (3)	Ca3—WA4C^{xix}	2.20 (6)
K2—WA3C^{viii}	2.65 (3)	Ca3—WA4C	2.20 (6)
K2—O4^{vi}	3.119 (2)	Ca3—WA4C^{xviii}	2.20 (6)
K2—O4	3.119 (2)	Ca3—WA2B^{xviii}	2.25 (3)
K2—O1^{ix}	3.2757 (14)	Ca3—WA2B	2.25 (3)
K2—O1^{x}	3.2757 (14)	Ca3—WA2B^{xix}	2.25 (3)
K2—O1^{vi}	3.2757 (14)	WA2B—WA4C	0.79 (5)
K2—O1	3.2757 (14)	WA2B—WA2B^{i}	1.43 (13)

O4—Si1—O1	107.26 (10)	WA1—Ca1—K2^{xx}	111.2 (3)
O4—Si1—O3	111.35 (7)	WA1B^{xiii}—Ca1—K2^{xx}	53.85 (15)
O1—Si1—O3	109.15 (9)	WA1B^{xiv}—Ca1—K2^{xx}	53.85 (15)
O4—Si1—O2	111.71 (9)	WA1B^{xv}—Ca1—K2^{xx}	121.5 (9)
O1—Si1—O2	110.49 (9)	Ca2^{xiv}—Ca1—K2^{xx}	68.8 (3)
O3—Si1—O2	106.89 (10)	WA5B^{xvi}—Ca1—K2^{xx}	101.7 (7)
O4—Si1—K1	139.79 (8)	WA5B^{i}—Ca1—K2^{xx}	11.0 (10)
O1—Si1—K1	112.17 (5)	WA5B^{xvii}—Ca1—K2^{xx}	101.7 (7)
O3—Si1—K1	62.49 (8)	WA1—Ca1—K2^{xxi}	111.2 (3)
O2—Si1—K1	46.40 (6)	WA1B^{xiii}—Ca1—K2^{xxi}	53.85 (15)
O4—Si1—K2	51.35 (8)	WA1B^{xiv}—Ca1—K2^{xxi}	121.5 (9)
O1—Si1—K2	56.98 (5)	WA1B^{xv}—Ca1—K2^{xxi}	53.85 (15)
O3—Si1—K2	116.62 (8)	Ca2^{xiv}—Ca1—K2^{xxi}	68.8 (3)
O2—Si1—K2	136.46 (7)	WA5B^{xvi}—Ca1—K2^{xxi}	101.7 (7)
K1—Si1—K2	168.859 (12)	WA5B^{i}—Ca1—K2^{xxi}	101.7 (7)
O6—Si2—O5	109.62 (14)	WA5B^{xvii}—Ca1—K2^{xxi}	11.0 (10)
O6—Si2—O1	107.77 (8)	K2^{xx}—Ca1—K2^{xxi}	107.7 (3)
O5—Si2—O1	110.88 (8)	WA1—Ca1—K2^{xxii}	111.2 (3)
O6—Si2—O1^{i}	107.77 (8)	WA1B^{xiii}—Ca1—K2^{xxii}	121.5 (9)
O5—Si2—O1^{i}	110.88 (8)	WA1B^{xiv}—Ca1—K2^{xxii}	53.85 (15)
O1—Si2—O1^{i}	109.83 (11)	WA1B^{xv}—Ca1—K2^{xxii}	53.85 (15)
O2^{ii}—K1—O2^{iii}	134.79 (3)	Ca2^{xiv}—Ca1—K2^{xxii}	68.8 (3)
O2^{ii}—K1—O2^{i}	83.49 (6)	WA5B^{xvi}—Ca1—K2^{xxii}	11.0 (10)
O2^{iii}—K1—O2^{i}	134.79 (3)	WA5B^{i}—Ca1—K2^{xxii}	101.7 (7)
O2^{ii}—K1—O2^{iv}	83.49 (6)	WA5B^{xvii}—Ca1—K2^{xxii}	101.7 (7)
O2^{iii}—K1—O2^{iv}	79.51 (8)	K2^{xx}—Ca1—K2^{xxii}	107.7 (3)
O2^{i}—K1—O2^{iv}	83.49 (6)	K2^{xxi}—Ca1—K2^{xxii}	107.7 (3)
O2^{ii}—K1—O2^{v}	79.51 (8)	Ca3—Ca2—WA3C^{xviii}	71.8 (9)
O2^{iii}—K1—O2^{v}	83.49 (6)	Ca3—Ca2—WA3C	71.8 (9)
O2^{i}—K1—O2^{v}	134.79 (3)	WA3C^{xviii}—Ca2—WA3C	110.7 (8)
O2^{iv}—K1—O2^{v}	134.79 (3)	Ca3—Ca2—WA3C^{xix}	71.8 (9)

O2^{ii}—K1—O2	134.79 (3)	WA3C^{xviii}—Ca2—WA3C^{xix}	110.7 (8)
O2^{iii}—K1—O2	83.48 (6)	WA3C—Ca2—WA3C^{xix}	110.7 (8)
O2^{i}—K1—O2	79.51 (8)	Ca3—Ca2—WA1B	125.2 (9)
O2^{iv}—K1—O2	134.79 (3)	WA3C^{xviii}—Ca2—WA1B	78.0 (6)
O2^{v}—K1—O2	83.48 (6)	WA3C—Ca2—WA1B	163.1 (14)
O2^{ii}—K1—O3^{iii}	171.12 (5)	WA3C^{xix}—Ca2—WA1B	78.0 (6)
O2^{iii}—K1—O3^{iii}	48.846 (16)	Ca3—Ca2—WA1B^{xix}	125.2 (8)
O2^{i}—K1—O3^{iii}	89.90 (4)	WA3C^{xviii}—Ca2—WA1B^{xix}	78.0 (6)
O2^{iv}—K1—O3^{iii}	89.90 (4)	WA3C—Ca2—WA1B^{xix}	78.0 (6)
O2^{v}—K1—O3^{iii}	109.37 (5)	WA3C^{xix}—Ca2—WA1B^{xix}	163.1 (14)
O2—K1—O3^{iii}	48.845 (15)	WA1B—Ca2—WA1B^{xix}	90.1 (12)
O2^{ii}—K1—O3^{i}	48.845 (16)	Ca3—Ca2—WA1B^{xviii}	125.2 (9)
O2^{iii}—K1—O3^{i}	171.12 (5)	WA3C^{xviii}—Ca2—WA1B^{xviii}	163.1 (14)
O2^{i}—K1—O3^{i}	48.846 (16)	WA3C—Ca2—WA1B^{xviii}	78.0 (6)
O2^{iv}—K1—O3^{i}	109.37 (5)	WA3C^{xix}—Ca2—WA1B^{xviii}	78.0 (6)
O2^{v}—K1—O3^{i}	89.90 (4)	WA1B—Ca2—WA1B^{xviii}	90.1 (12)
O2—K1—O3^{i}	89.90 (4)	WA1B^{xix}—Ca2—WA1B^{xviii}	90.1 (12)
O3^{iii}—K1—O3^{i}	129.173 (17)	Ca3—Ca2—WA2B^{i}	44.0 (8)
O2^{ii}—K1—O3^{ii}	48.846 (15)	WA3C^{xviii}—Ca2—WA2B^{i}	56.3 (6)
O2^{iii}—K1—O3^{ii}	89.90 (4)	WA3C—Ca2—WA2B^{i}	115.8 (13)
O2^{i}—K1—O3^{ii}	109.37 (5)	WA3C^{xix}—Ca2—WA2B^{i}	56.3 (6)
O2^{iv}—K1—O3^{ii}	48.845 (15)	WA1B—Ca2—WA2B^{i}	81.2 (10)
O2^{v}—K1—O3^{ii}	89.90 (4)	WA1B^{xix}—Ca2—WA2B^{i}	134.3 (4)
O2—K1—O3^{ii}	171.12 (5)	WA1B^{xviii}—Ca2—WA2B^{i}	134.3 (4)
O3^{iii}—K1—O3^{ii}	129.173 (17)	Ca3—Ca2—WA2B^{vii}	44.0 (8)
O3^{i}—K1—O3^{ii}	96.03 (4)	WA3C^{xviii}—Ca2—WA2B^{vii}	115.8 (13)
O2^{ii}—K1—O3^{v}	89.90 (4)	WA3C—Ca2—WA2B^{vii}	56.3 (6)
O2^{iii}—K1—O3^{v}	48.845 (15)	WA3C^{xix}—Ca2—WA2B^{vii}	56.3 (6)
O2^{i}—K1—O3^{v}	171.12 (5)	WA1B—Ca2—WA2B^{vii}	134.3 (4)
O2^{iv}—K1—O3^{v}	89.90 (4)	WA1B^{xix}—Ca2—WA2B^{vii}	134.3 (5)
O2^{v}—K1—O3^{v}	48.846 (15)	WA1B^{xviii}—Ca2—WA2B^{vii}	81.2 (10)

O2—K1—O3^{v}	109.37 (5)	WA2B^{i}—Ca2—WA2B^{vii}	74.0 (12)
O3^{iii}—K1—O3^{v}	96.03 (4)	Ca3—Ca2—WA2B^{xi}	44.0 (8)
O3^{i}—K1—O3^{v}	129.173 (17)	WA3C^{xviii}—Ca2—WA2B^{xi}	56.3 (6)
O3^{ii}—K1—O3^{v}	61.75 (6)	WA3C—Ca2—WA2B^{xi}	56.3 (6)
O2^{ii}—K1—O3^{iv}	109.37 (5)	WA3C^{xix}—Ca2—WA2B^{xi}	115.8 (13)
O2^{iii}—K1—O3^{iv}	89.90 (4)	WA1B—Ca2—WA2B^{xi}	134.3 (4)
O2^{i}—K1—O3^{iv}	48.845 (15)	WA1B^{xix}—Ca2—WA2B^{xi}	81.2 (10)
O2^{iv}—K1—O3^{iv}	48.846 (16)	WA1B^{xviii}—Ca2—WA2B^{xi}	134.3 (4)
O2^{v}—K1—O3^{iv}	171.12 (5)	WA2B^{i}—Ca2—WA2B^{xi}	74.0 (12)
O2—K1—O3^{iv}	89.90 (4)	WA2B^{vii}—Ca2—WA2B^{xi}	74.0 (12)
O3^{iii}—K1—O3^{iv}	61.75 (6)	Ca3—Ca2—Ca1^{xiv}	180.0
O3^{i}—K1—O3^{iv}	96.03 (4)	WA3C^{xviii}—Ca2—Ca1^{xiv}	108.2 (9)
O3^{ii}—K1—O3^{iv}	96.03 (4)	WA3C—Ca2—Ca1^{xiv}	108.2 (9)
O3^{v}—K1—O3^{iv}	129.173 (17)	WA3C^{xix}—Ca2—Ca1^{xiv}	108.2 (9)
O2^{ii}—K1—O3	89.90 (4)	WA1B—Ca2—Ca1^{xiv}	54.8 (8)
O2^{iii}—K1—O3	109.37 (5)	WA1B^{xix}—Ca2—Ca1^{xiv}	54.8 (8)
O2^{i}—K1—O3	89.90 (4)	WA1B^{xviii}—Ca2—Ca1^{xiv}	54.8 (8)
O2^{iv}—K1—O3	171.12 (5)	WA2B^{i}—Ca2—Ca1^{xiv}	136.0 (8)
O2^{v}—K1—O3	48.845 (16)	WA2B^{vii}—Ca2—Ca1^{xiv}	136.0 (8)
O2—K1—O3	48.845 (16)	WA2B^{xi}—Ca2—Ca1^{xiv}	136.0 (8)
O3^{iii}—K1—O3	96.03 (4)	Ca3^{i}—Ca3—WA2B^{vii}	91 (2)
O3^{i}—K1—O3	61.75 (6)	Ca3^{i}—Ca3—WA2B^{i}	91 (2)
O3^{ii}—K1—O3	129.173 (17)	WA2B^{vii}—Ca3—WA2B^{i}	119.97 (16)
O3^{v}—K1—O3	96.03 (4)	Ca3^{i}—Ca3—WA2B^{xi}	91 (2)
O3^{iv}—K1—O3	129.173 (17)	WA2B^{vii}—Ca3—WA2B^{xi}	119.96 (19)
WA5B^{vi}—K2—WA5B	180.0	WA2B^{i}—Ca3—WA2B^{xi}	119.96 (16)
WA5B^{vi}—K2—WA3C^{vii}	36 (3)	Ca3^{i}—Ca3—Ca2	180.0
WA5B—K2—WA3C^{vii}	144 (3)	WA2B^{vii}—Ca3—Ca2	89 (2)
WA5B^{vi}—K2—WA3C^{viii}	144 (3)	WA2B^{i}—Ca3—Ca2	89 (2)
WA5B—K2—WA3C^{viii}	36 (3)	WA2B^{xi}—Ca3—Ca2	89 (2)
WA3C^{vii}—K2—WA3C^{viii}	180.0	Ca3^{i}—Ca3—WA4C^{xix}	72.0 (7)

WA5B^{vi}—K2—O4^{vi}	89.999 (12)	WA2B^{vii}—Ca3—WA4C^{xix}	118.8 (7)
WA5B—K2—O4^{vi}	89.999 (9)	WA2B^{i}—Ca3—WA4C^{xix}	118.8 (7)
WA3C^{vii}—K2—O4^{vi}	90.000 (1)	WA2B^{xi}—Ca3—WA4C^{xix}	19 (3)
WA3C^{viii}—K2—O4^{vi}	90.000 (1)	Ca2—Ca3—WA4C^{xix}	108.0 (7)
WA5B^{vi}—K2—O4	90.001 (12)	Ca3^{i}—Ca3—WA4C	72.0 (7)
WA5B—K2—O4	90.001 (9)	WA2B^{vii}—Ca3—WA4C	118.8 (7)
WA3C^{vii}—K2—O4	90.000 (1)	WA2B^{i}—Ca3—WA4C	19 (3)
WA3C^{viii}—K2—O4	90.000 (1)	WA2B^{xi}—Ca3—WA4C	118.8 (7)
O4^{vi}—K2—O4	180.0	Ca2—Ca3—WA4C	108.0 (7)
WA5B^{vi}—K2—O1^{ix}	89 (2)	WA4C^{xix}—Ca3—WA4C	110.9 (6)
WA5B—K2—O1^{ix}	91 (2)	Ca3^{i}—Ca3—WA4C^{xviii}	72.0 (7)
WA3C^{vii}—K2—O1^{ix}	62.9 (2)	WA2B^{vii}—Ca3—WA4C^{xviii}	19 (3)
WA3C^{viii}—K2—O1^{ix}	117.1 (2)	WA2B^{i}—Ca3—WA4C^{xviii}	118.8 (7)
O4^{vi}—K2—O1^{ix}	48.47 (2)	WA2B^{xi}—Ca3—WA4C^{xviii}	118.8 (7)
O4—K2—O1^{ix}	131.53 (2)	Ca2—Ca3—WA4C^{xviii}	108.0 (7)
WA5B^{vi}—K2—O1^{x}	91 (2)	WA4C^{xix}—Ca3—WA4C^{xviii}	110.9 (6)
WA5B—K2—O1^{x}	89 (2)	WA4C—Ca3—WA4C^{xviii}	110.9 (6)
WA3C^{vii}—K2—O1^{x}	117.1 (2)	Ca3^{i}—Ca3—WA2B^{xviii}	51.7 (17)
WA3C^{viii}—K2—O1^{x}	62.9 (2)	WA2B^{vii}—Ca3—WA2B^{xviii}	39 (4)
O4^{vi}—K2—O1^{x}	131.53 (2)	WA2B^{i}—Ca3—WA2B^{xviii}	113.9 (10)
O4—K2—O1^{x}	48.47 (2)	WA2B^{xi}—Ca3—WA2B^{xviii}	113.8 (10)
O1^{ix}—K2—O1^{x}	180.0	Ca2—Ca3—WA2B^{xviii}	128.3 (17)
WA5B^{vi}—K2—O1^{vi}	91 (2)	WA4C^{xix}—Ca3—WA2B^{xviii}	100.4 (13)
WA5B—K2—O1^{vi}	89 (2)	WA4C—Ca3—WA2B^{xviii}	100.4 (13)
WA3C^{vii}—K2—O1^{vi}	117.1 (2)	WA4C^{xviii}—Ca3—WA2B^{xviii}	20.3 (14)
WA3C^{viii}—K2—O1^{vi}	62.9 (2)	Ca3^{i}—Ca3—WA2B	51.7 (17)
O4^{vi}—K2—O1^{vi}	48.47 (2)	WA2B^{vii}—Ca3—WA2B	113.8 (10)
O4—K2—O1^{vi}	131.53 (2)	WA2B^{i}—Ca3—WA2B	39 (4)
O1^{ix}—K2—O1^{vi}	96.93 (4)	WA2B^{xi}—Ca3—WA2B	113.8 (10)
O1^{x}—K2—O1^{vi}	83.07 (4)	Ca2—Ca3—WA2B	128.3 (18)
WA5B^{vi}—K2—O1	89 (2)	WA4C^{xix}—Ca3—WA2B	100.4 (13)

WA5B—K2—O1	91 (2)	WA4C—Ca3—WA2B	20.3 (14)
WA3C^{vii}—K2—O1	62.9 (2)	WA4C^{xviii}—Ca3—WA2B	100.4 (13)
WA3C^{viii}—K2—O1	117.1 (2)	WA2B^{xviii}—Ca3—WA2B	86 (3)
O4^{vi}—K2—O1	131.53 (2)	Ca3^{i}—Ca3—WA2B^{xix}	51.7 (17)
O4—K2—O1	48.47 (2)	WA2B^{vii}—Ca3—WA2B^{xix}	113.8 (10)
O1^{ix}—K2—O1	83.07 (4)	WA2B^{i}—Ca3—WA2B^{xix}	113.8 (10)
O1^{x}—K2—O1	96.93 (4)	WA2B^{xi}—Ca3—WA2B^{xix}	39 (4)
O1^{vi}—K2—O1	180.0	Ca2—Ca3—WA2B^{xix}	128.3 (17)
WA5B^{vi}—K2—WA1B^{xi}	30.4 (10)	WA4C^{xix}—Ca3—WA2B^{xix}	20.3 (14)
WA5B—K2—WA1B^{xi}	149.6 (10)	WA4C—Ca3—WA2B^{xix}	100.4 (13)
WA3C^{vii}—K2—WA1B^{xi}	52.9 (5)	WA4C^{xviii}—Ca3—WA2B^{xix}	100.4 (13)
WA3C^{viii}—K2—WA1B^{xi}	127.1 (5)	WA2B^{xviii}—Ca3—WA2B^{xix}	86 (3)
O4^{vi}—K2—WA1B^{xi}	61.1 (4)	WA2B—Ca3—WA2B^{xix}	86 (3)
O4—K2—WA1B^{xi}	118.9 (4)	Si1—O1—Si2	143.22 (10)
O1^{ix}—K2—WA1B^{xi}	77.4 (5)	Si1—O1—K2	98.31 (6)
O1^{x}—K2—WA1B^{xi}	102.6 (5)	Si2—O1—K2	115.19 (6)
O1^{vi}—K2—WA1B^{xi}	65.0 (4)	Si1—O2—Si1^{xxiii}	140.86 (13)
O1—K2—WA1B^{xi}	115.0 (4)	Si1—O2—K1	109.53 (6)
WA5B^{vi}—K2—WA1B^{xii}	149.6 (10)	Si1^{xxiii}—O2—K1	109.53 (6)
WA5B—K2—WA1B^{xii}	30.4 (10)	Si1^{xxiv}—O3—Si1	143.86 (12)
WA3C^{vii}—K2—WA1B^{xii}	127.1 (5)	Si1^{xxiv}—O3—K1	92.05 (8)
WA3C^{viii}—K2—WA1B^{xii}	52.9 (5)	Si1—O3—K1	92.05 (8)
O4^{vi}—K2—WA1B^{xii}	118.9 (4)	Si1—O4—Si1^{x}	150.91 (16)
O4—K2—WA1B^{xii}	61.1 (4)	Si1—O4—K2	104.55 (8)
O1^{ix}—K2—WA1B^{xii}	102.6 (5)	Si1^{x}—O4—K2	104.55 (8)
O1^{x}—K2—WA1B^{xii}	77.4 (5)	Si2—O5—Si2^{xxiii}	150.3 (2)
O1^{vi}—K2—WA1B^{xii}	115.0 (4)	Si2—O6—Si2^{ix}	171.1 (2)
O1—K2—WA1B^{xii}	65.0 (4)	Ca2—WA1B—Ca1^{xiv}	72.5 (10)
WA1B^{xi}—K2—WA1B^{xii}	180.0	Ca2—WA1B—K2^{xxv}	90.2 (4)
WA1—Ca1—WA1B^{xiii}	127.3 (7)	Ca1^{xiv}—WA1B—K2^{xxv}	91.7 (5)
WA1—Ca1—WA1B^{xiv}	127.3 (7)	Ca2—WA1B—K2^{xx}	90.2 (4)

$WA1B^{xiii}—Ca1—WA1B^{xiv}$	87.0 (11)	$Ca1^{xiv}—WA1B—K2^{xx}$	91.7 (5)
$WA1—Ca1—WA1B^{xv}$	127.3 (7)	$K2^{xxv}—WA1B—K2^{xx}$	176.6 (10)
$WA1B^{xiii}—Ca1—WA1B^{xv}$	87.0 (11)	$WA4C—WA2B—WA2B^{i}$	24 (5)
$WA1B^{xiv}—Ca1—WA1B^{xv}$	87.0 (11)	$WA4C—WA2B—Ca3^{i}$	113 (7)
$WA1—Ca1—Ca2^{xiv}$	180.0	$WA2B^{i}—WA2B—Ca3^{i}$	89 (2)
$WA1B^{xiii}—Ca1—Ca2^{xiv}$	52.7 (7)	WA4C—WA2B—Ca3	76 (6)
$WA1B^{xiv}—Ca1—Ca2^{xiv}$	52.7 (7)	$WA2B^{i}—WA2B—Ca3$	51.7 (18)
$WA1B^{xv}—Ca1—Ca2^{xiv}$	52.7 (7)	$Ca3^{i}—WA2B—Ca3$	37.2 (11)
$WA1—Ca1—WA5B^{xvi}$	122.2 (11)	$WA4C—WA2B—Ca2^{i}$	160 (6)
$WA1B^{xiii}—Ca1—WA5B^{xvi}$	110.5 (15)	$WA2B^{i}—WA2B—Ca2^{i}$	136.0 (8)
$WA1B^{xiv}—Ca1—WA5B^{xvi}$	48.7 (5)	$Ca3^{i}—WA2B—Ca2^{i}$	47.1 (16)
$WA1B^{xv}—Ca1—WA5B^{xvi}$	48.7 (5)	$Ca3—WA2B—Ca2^{i}$	84.3 (13)
$Ca2^{xiv}—Ca1—WA5B^{xvi}$	57.8 (11)	$K2—WA5B—Ca2^{xxvi}$	167 (4)
$WA1—Ca1—WA5B^{i}$	122.2 (11)	$K2—WA5B—Ca1^{i}$	139 (4)
$WA1B^{xiii}—Ca1—WA5B^{i}$	48.7 (5)	$Ca2^{xxvi}—WA5B—Ca1^{i}$	53.8 (9)
$WA1B^{xiv}—Ca1—WA5B^{i}$	48.7 (5)	Ca2—WA3C—Ca3	50.1 (8)
$WA1B^{xv}—Ca1—WA5B^{i}$	110.5 (15)	$Ca2—WA3C—K2^{xxvii}$	116.7 (10)
$Ca2^{xiv}—Ca1—WA5B^{i}$	57.8 (11)	$Ca3—WA3C—K2^{xxvii}$	166.8 (8)
$WA5B^{xvi}—Ca1—WA5B^{i}$	94.3 (15)	$WA2B^{i}—WA4C—WA2B$	131 (10)
$WA1—Ca1—WA5B^{xvii}$	122.2 (11)	$WA2B^{i}—WA4C—Ca3^{i}$	84 (5)
$WA1B^{xiii}—Ca1—WA5B^{xvii}$	48.7 (5)	$WA2B—WA4C—Ca3^{i}$	48 (5)
$WA1B^{xiv}—Ca1—WA5B^{xvii}$	110.5 (15)	$WA2B^{i}—WA4C—Ca3$	48 (5)
$WA1B^{xv}—Ca1—WA5B^{xvii}$	48.7 (5)	WA2B—WA4C—Ca3	84 (5)
$Ca2^{xiv}—Ca1—WA5B^{xvii}$	57.8 (11)	$Ca3^{i}—WA4C—Ca3$	36.1 (13)
$WA5B^{xvi}—Ca1—WA5B^{xvii}$	94.3 (15)	$Ca1^{i}—WA1—Ca1$	180.0
$WA5B^{i}—Ca1—WA5B^{xvii}$	94.3 (15)		

Symmetry code(s): (i) *x*, *y*, −*z*+1/2; (ii) −*y*+1, *x*−*y*+2, −*z*+1/2; (iii) −*x*+*y*−1, −*x*+1, *z*; (iv) −*x*+*y*−1, −*x*+1, −*z*+1/2; (v) −*y*+1, *x*−*y*+2, *z*; (vi) −*x*+1, −*y*+2, −*z*; (vii) −*x*+*y*, −*x*+2, −*z*+1/2; (viii) *x*−*y*+1, *x*, *z*−1/2; (ix) −*x*+*y*, *y*, *z*; (x) *x*−*y*+1, −*y*+2, −*z*; (xi) −*y*+2, *x*−*y*+2, −*z*+1/2; (xii) *y*−1, −*x*+*y*, *z*−1/2; (xiii) *y*−1, −*x*+*y*, −*z*+1; (xiv) −*x*+1, −*y*+2, −*z*+1; (xv) *x*−*y*+1, *x*, −*z*+1; (xvi) −*x*+*y*, −*x*+1, −*z*+1/2; (xvii) −*y*+1,

$x-y+1$, $-z+1/2$; (xviii) $-x+y$, $-x+2$, z; (xix) $-y+2$, $x-y+2$, z; (xx) $-x+1$, $-y+2$, $z+1/2$; (xxi) $y-1$, $-x+y$, $z+1/2$; (xxii) $x-y+1$, x, $z+1/2$; (xxiii) $-y+1$, $-x+1$, z; (xxiv) x, $x-y+2$, z; (xxv) $x-y+1$, $x+1$, $z+1/2$; (xxvi) $-x+1$, $-y+2$, $z-1/2$; (xxvii) y, $-x+y+1$, $z+1/2$.

Table S4. Site symmetry (s.s.), site partition, final atomic coordinates, and atomic displacement parameters (Å^2) for the erionite from Tuzköy.

site	s.s.	site partition	x/a	y/b	z/c	U_{eq}	U^{11}	U^{22}	U^{33}	U^{23}	U^{13}	U^{12}
Si1	1	0.860(15)	0.23398(4)	0.99978(3)	0.10458(3)	0.01727(18)	0.0224(3)	0.0174(2)	0.0129(2)	0.00090(11)	-0.00166(13)	0.01061(16)
Al1	1	0.140(15)	0.23398(4)	0.99978(3)	0.10458(3)	0.01727(18)	0.0224(3)	0.0174(2)	0.0129(2)	0.00090(11)	-0.00166(13)	0.01061(16)
Si2	m..	0.66(3)	0.33174(4)	0.90620(5)	0.25	0.0148(2)	0.0135(3)	0.0172(3)	0.0157(3)	0	0	0.0091(2)
Al2	m..	0.34(3)	0.33174(4)	0.90620(5)	0.25	0.0148(2)	0.0135(3)	0.0172(3)	0.0157(3)	0	0	0.0091(2)
K1	-6m2	1	0	1	0.25	0.0341(3)	0.0355(5)	0.0355(5)	0.0313(7)	0	0	0.0178(2)
K2	.2/m.	0.105	0.5	1	0	0.152(15)	0.082(11)	0.23(4)	0.19(3)	-0.06(3)	-0.032(14)	0.12(2)
Ca1	3m.	0.39(3)	0.333333	0.666667	0.4016(14)	0.29(2)	0.31(2)	0.31(2)	0.25(2)	0	0	0.155(12)
Ca2	3m.	0.2059(12)	0.666667	1.333333	0.4186(13)	0.132(6)	0.084(5)	0.084(5)	0.228(19)	0	0	0.042(2)
Na2	3m.	0.033	0.666667	1.333333	0.4186(13)	0.132(6)	0.084(5)	0.084(5)	0.228(19)	0	0	0.042(2)
Ca3	3m.	0.06(2)	0.666667	1.333333	0.2951(12)	0.084(6)	0.038(4)	0.038(4)	0.176(17)	0	0	0.0189(19)
Mg3	3m.	0.15(3)	0.666667	1.333333	0.2951(12)	0.084(6)	0.038(4)	0.038(4)	0.176(17)	0	0	0.0189(19)
O1	1	1	0.32241(13)	0.97215(14)	0.16106(9)	0.0365(3)	0.0393(7)	0.0485(8)	0.0304(6)	0.0107(6)	-0.0008(6)	0.0285(6)
O2	.m.	1	0.09775(9)	0.90225(9)	0.12617(13)	0.0318(4)	0.0262(5)	0.0262(5)	0.0352(9)	-0.0019(4)	0.0019(4)	0.0071(7)
O3	.m.	1	0.25212(19)	1.12606(9)	0.13523(11)	0.0336(4)	0.0523(11)	0.0293(6)	0.0270(8)	-0.0035(4)	-0.0070(8)	0.0262(6)
O4	.2.	1	0.26497(18)	1	0	0.0325(4)	0.0421(8)	0.0375(10)	0.0164(7)	-0.0004(6)	-0.0002(3)	0.0187(5)
O5	mm2	1	0.23098(12)	0.76902(12)	0.25	0.0361(6)	0.0241(8)	0.0241(8)	0.0548(17)	0	0	0.0082(10)
O6	mm2	1	0.45857(13)	0.9171(3)	0.25	0.0346(6)	0.0233(8)	0.0439(15)	0.0434(14)	0	0	0.0220(8)
OW1	1	0.62(4)	0.506(2)	1.2529(10)	0.5049(17)	0.36(2)	0.17(2)	0.47(4)	0.34(3)	0.024(8)	0.048(16)	0.087(11)
OW2	.m.	0.35(6)	0.513(3)	1.2565(13)	0.203(4)	0.123(15)	0.066(11)	0.138(13)	0.14(3)	-0.014(7)	-0.027(15)	0.033(6)
OW3	.m.	0.61(4)	0.837(2)	1.4186(10)	0.3759(15)	0.244(16)	0.29(3)	0.223(16)	0.24(3)	0.049(9)	0.098(19)	0.147(14)
OW4	m..	0.30(10)	0.485(5)	1.242(3)	0.25	0.116(14)	0.10(2)	0.16(2)	0.06(3)	0	0	0.049(11)
OW5	-6m2	0.12(4)	0.333333	0.666667	0.25	0.14(6)	0.09(5)	0.09(5)	0.23(11)	0	0	0.04(3)

OW6	1	0.50(4)	0.4487(18)	0.897(4)	-0.012(4)	0.39(4)	0.177(19)	0.47(6)	0.63(8)	-0.09(5)	-0.04(3)	0.24(3)
